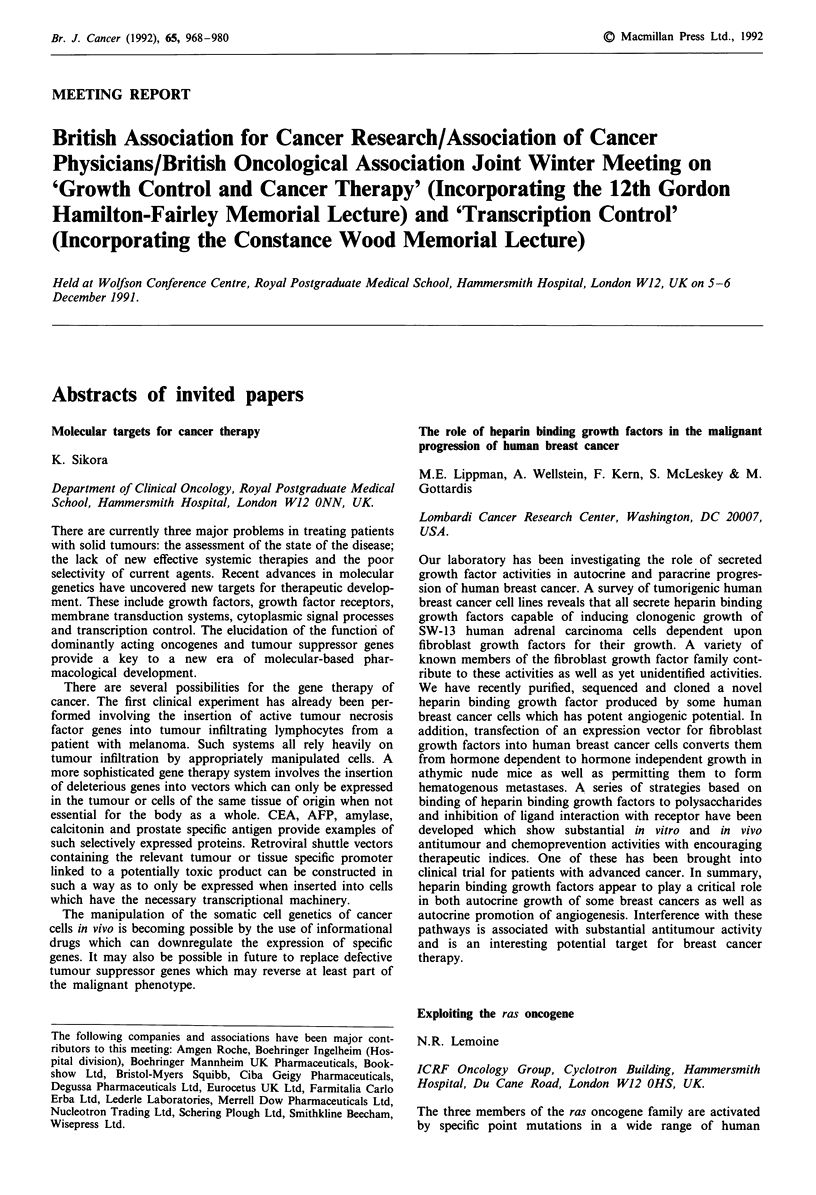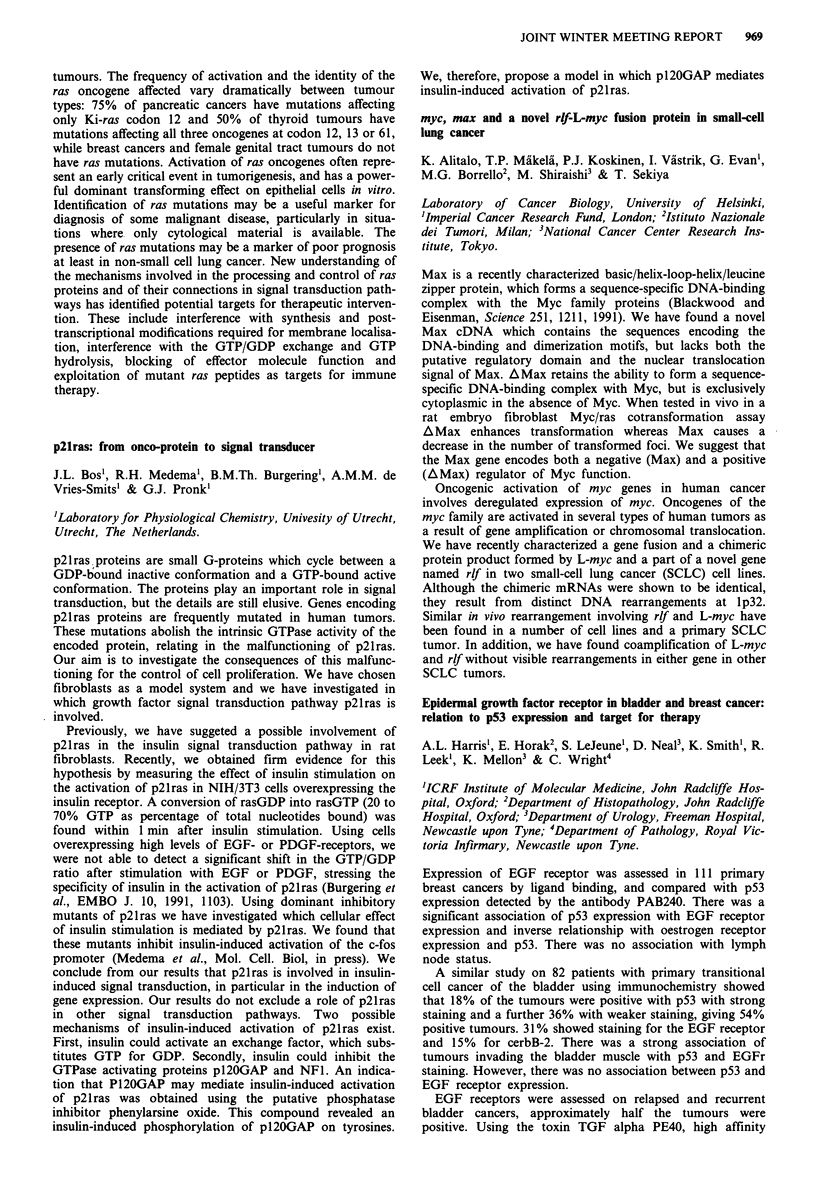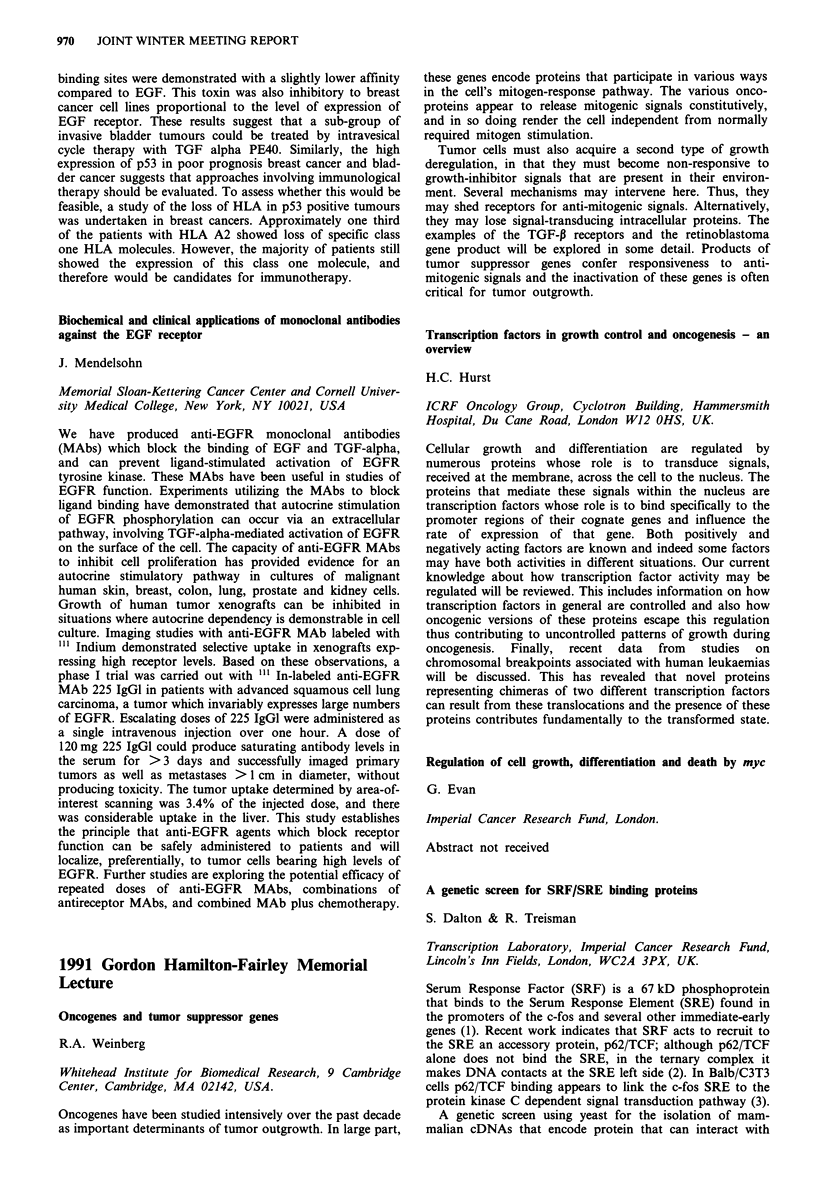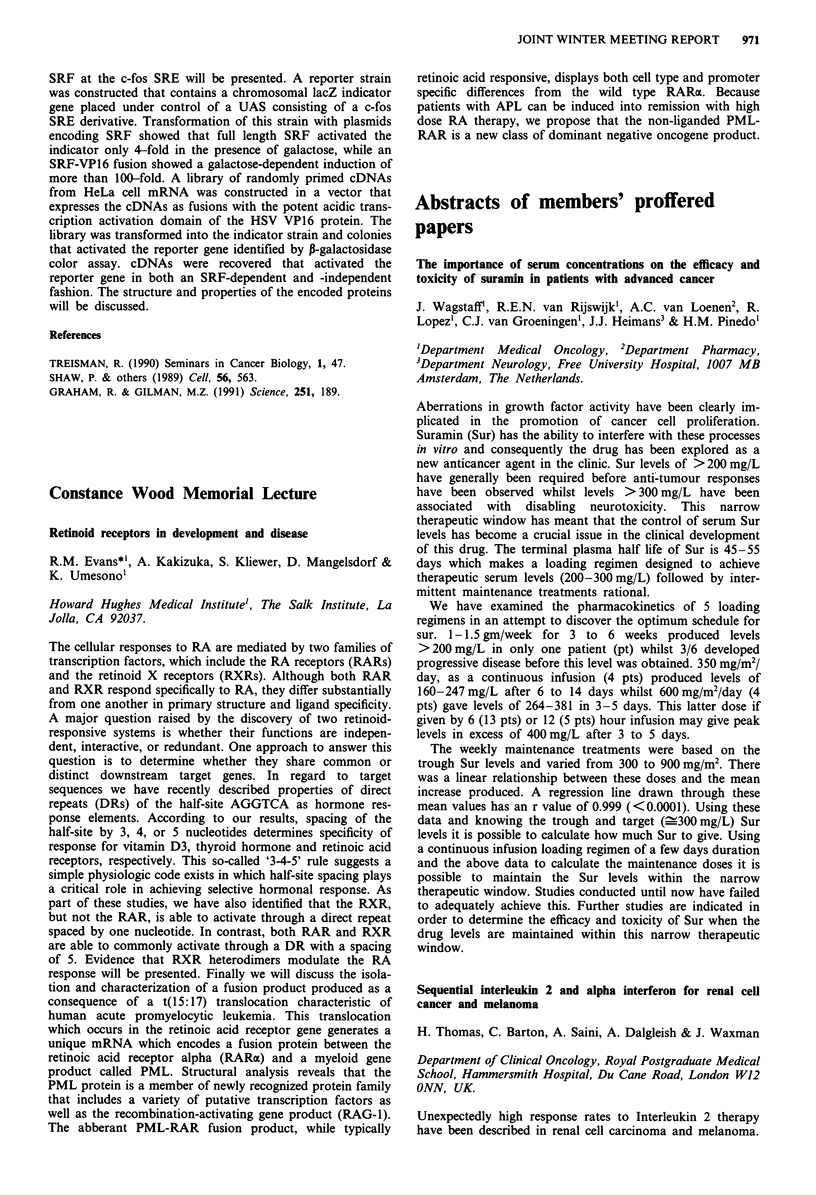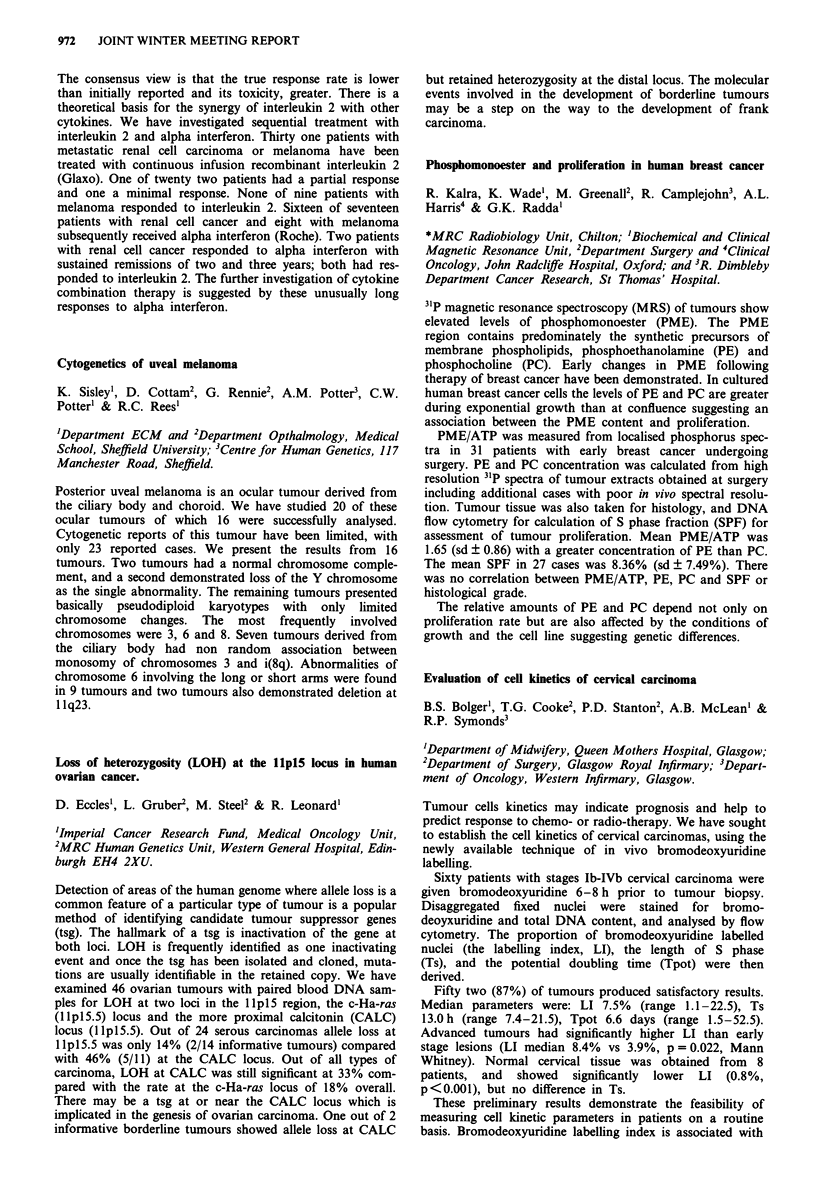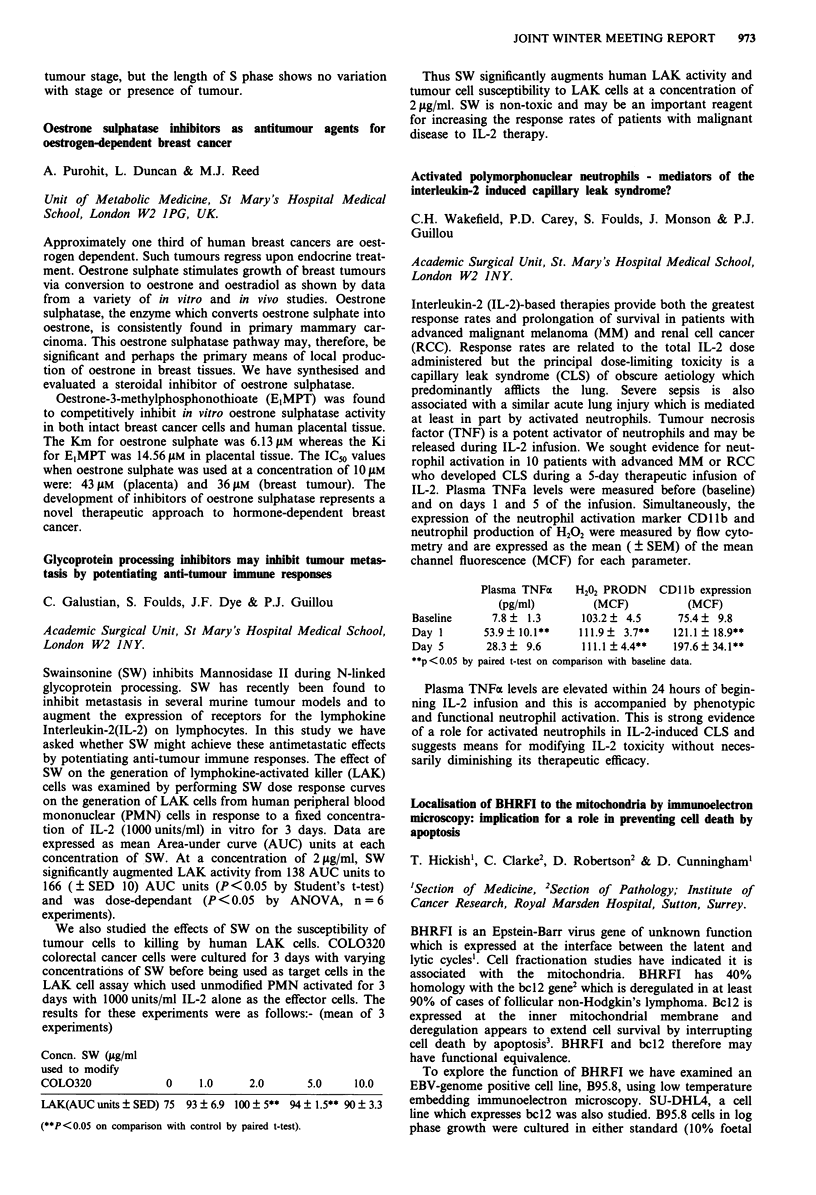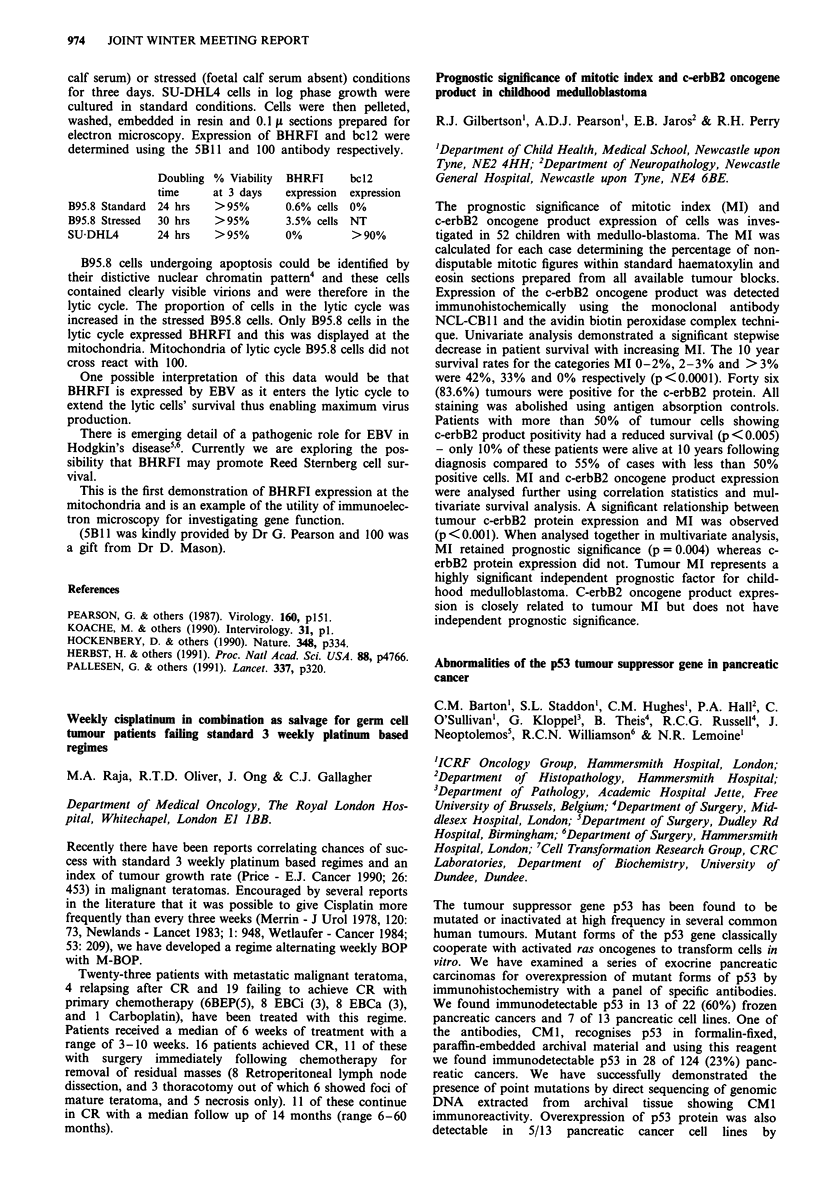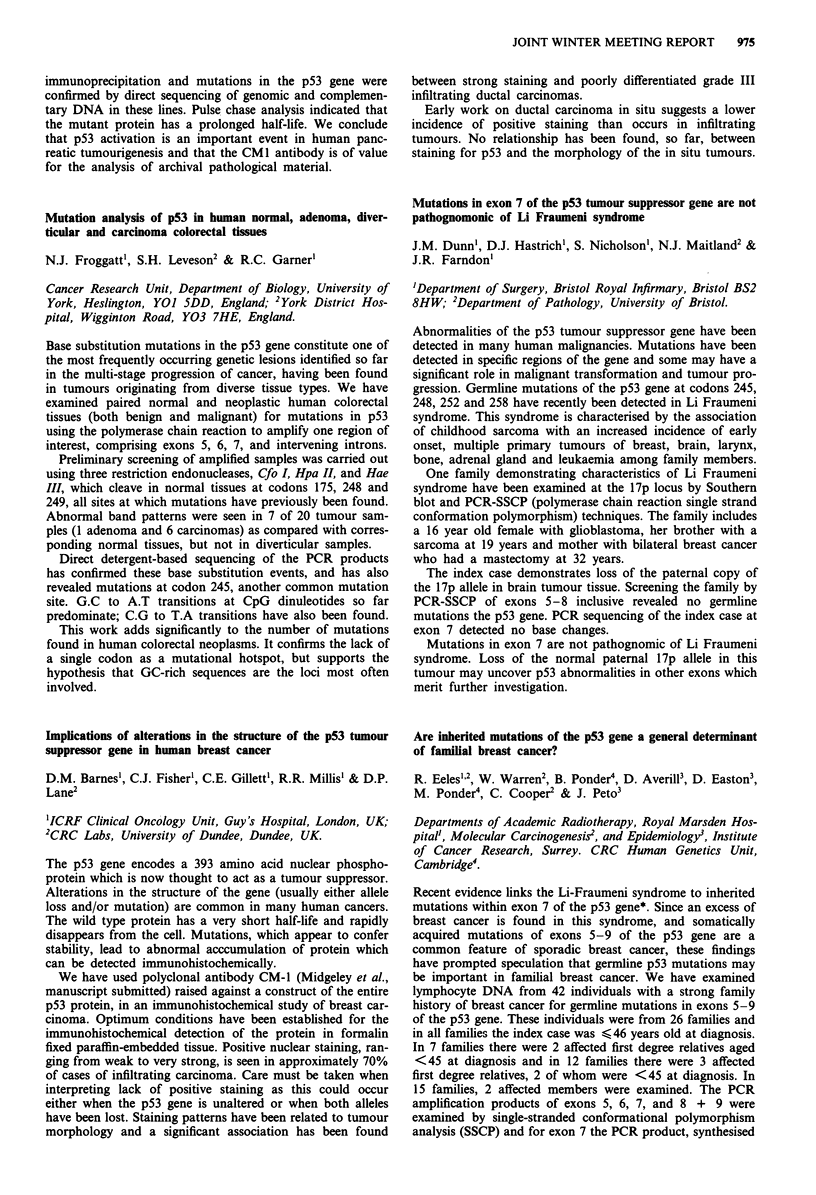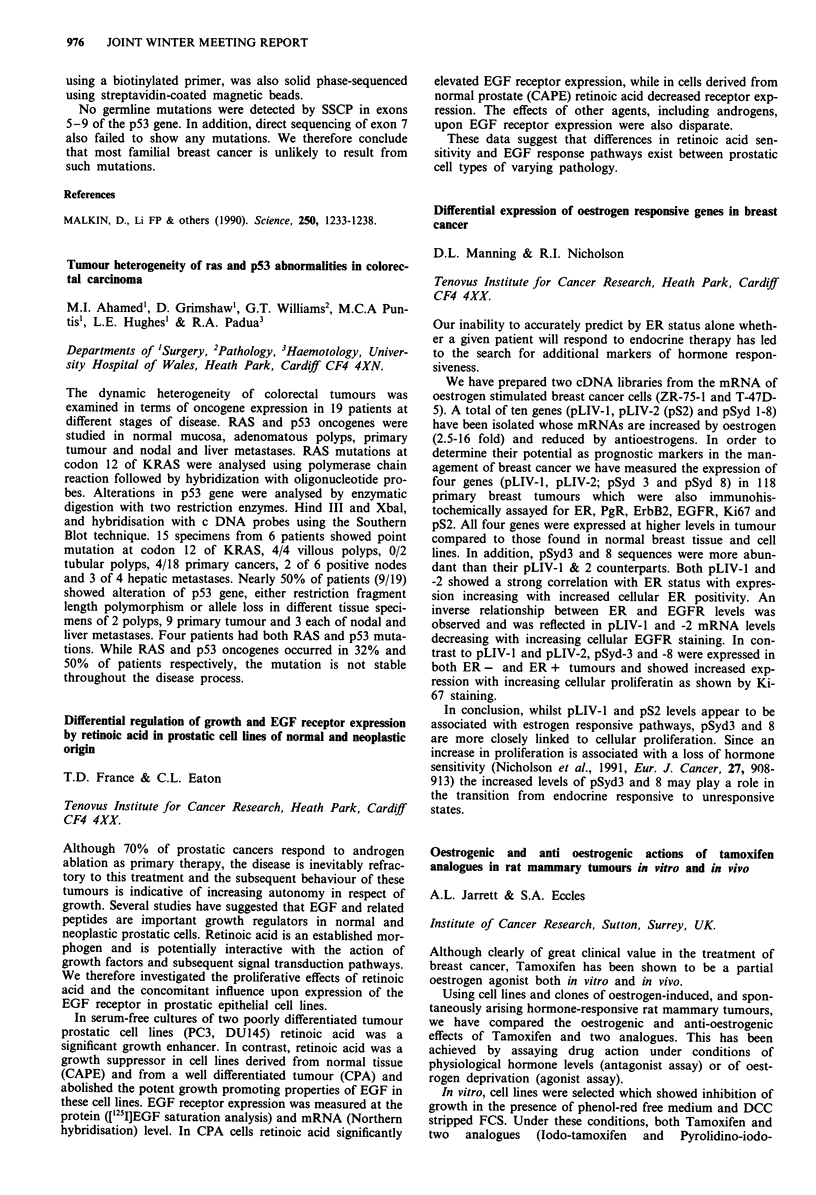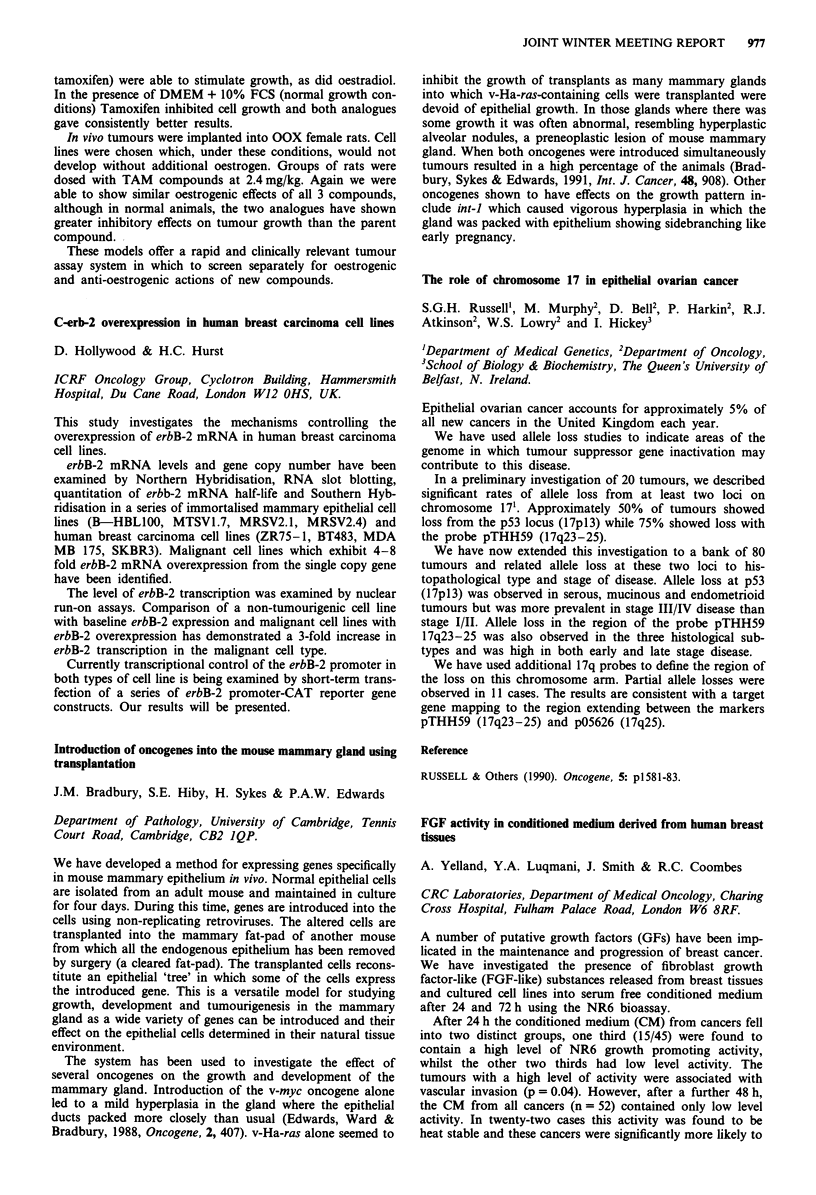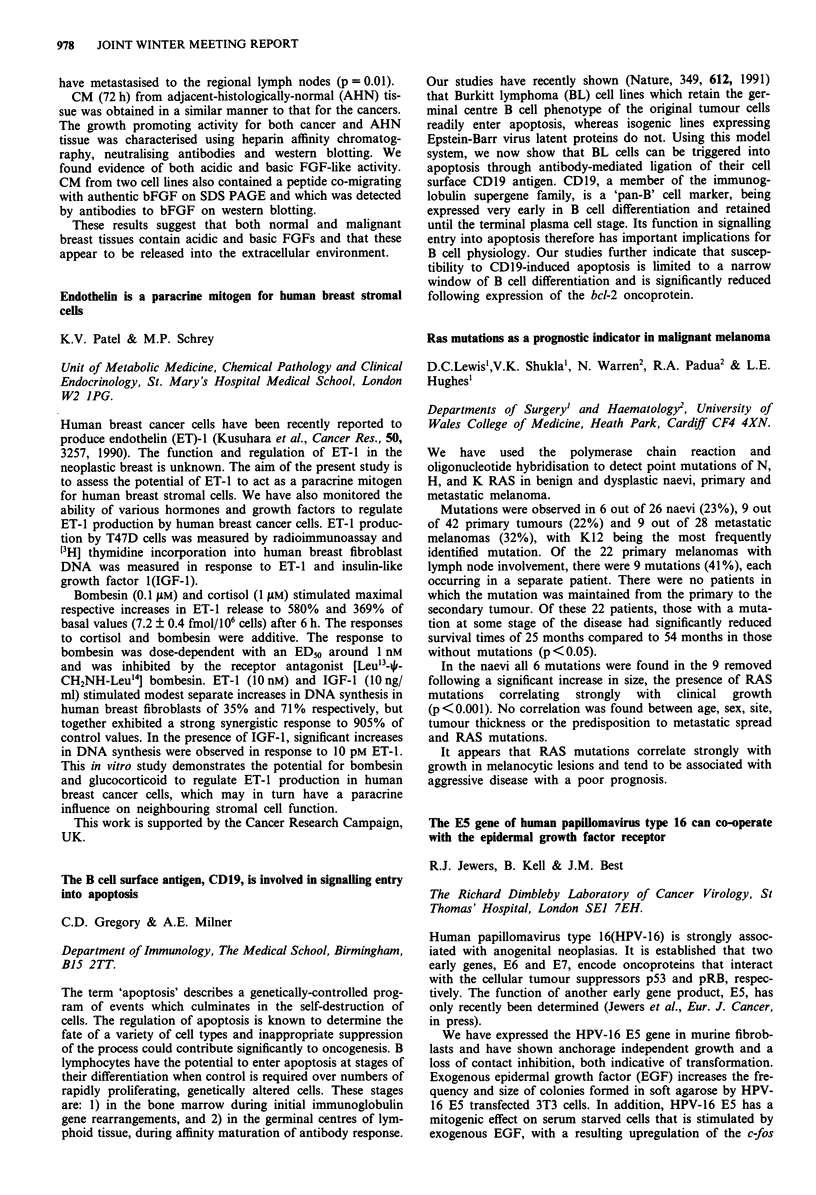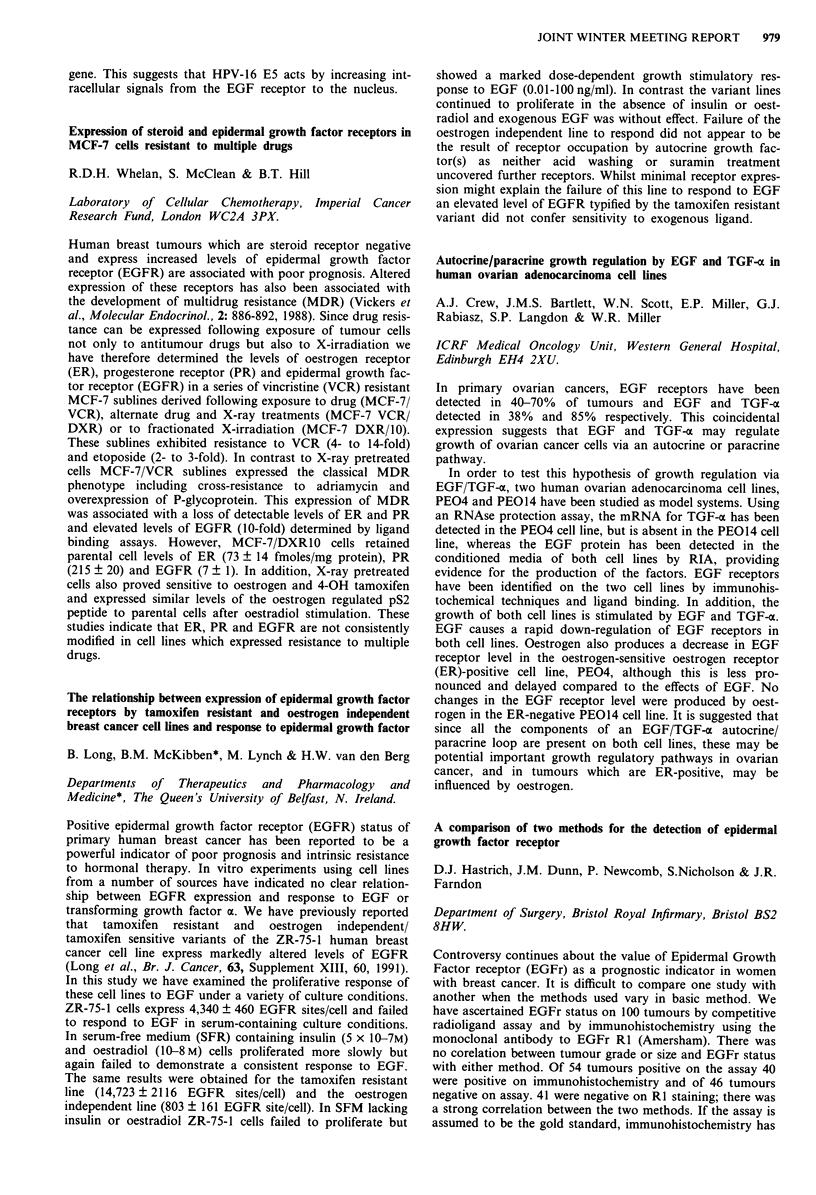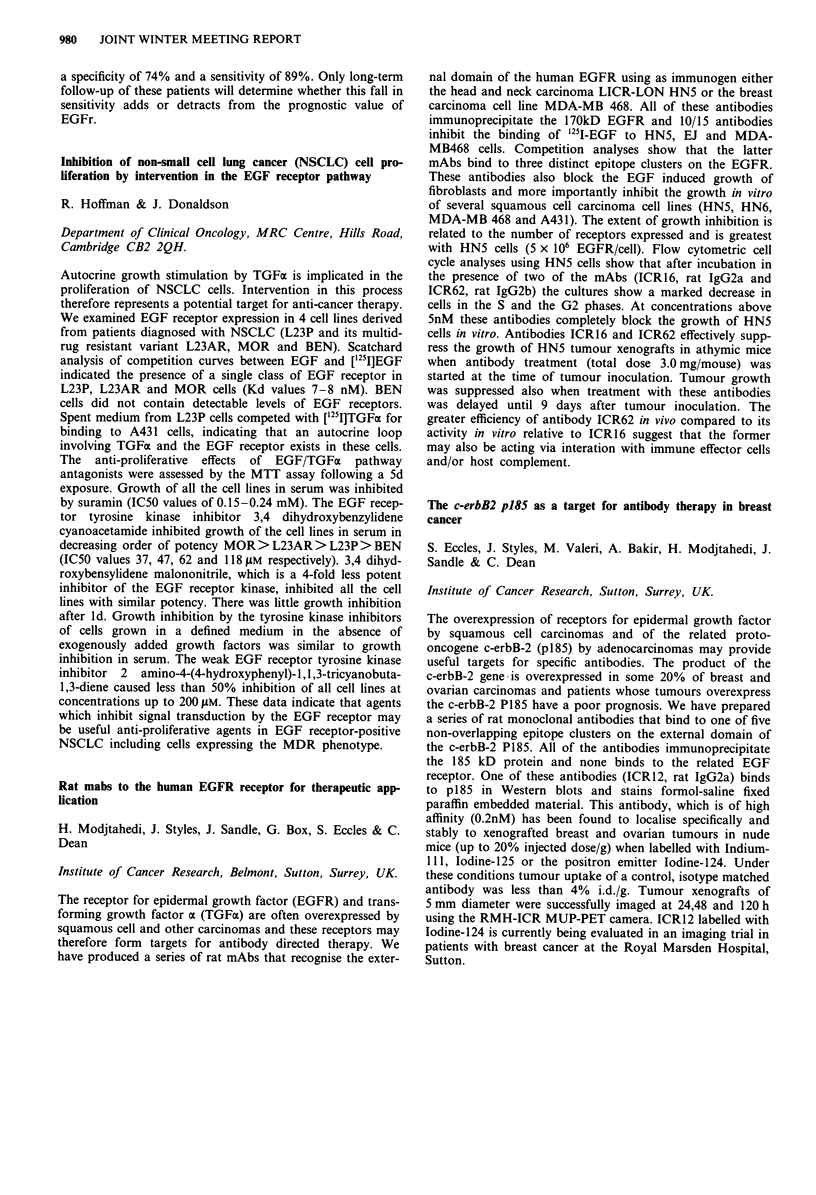# British Association for Cancer Research/Association of Cancer Physicians/British Oncological Association

**Published:** 1992-06

**Authors:** 


Br. J. Cancer (1992), 65, 968-980 © Macmillan Press Ltd., 1992

MEETING REPORT

### British Association for Cancer Research/Association of Cancer Physicians/British Oncological Association Joint Winter Meeting on 'Growth Control and Cancer Therapy' (Incorporating the 12th Gordon Hamilton-Fairley Memorial Lecture) and 'Transcription Control' (Incorporating the Constance Wood Memorial Lecture)

*Held at Wolfson Conference Centre, Royal Postgraduate Medical School, Hammersmith Hospital, London W12, UK on 5-6 December 1991.*

### Abstracts of invited papers

### Molecular targets for cancer therapy

K. Sikora

*Department of Clinical Oncology, Royal Postgraduate Medical School, Hammersmith Hospital, London W12 0NN, UK.*

There are currently three major problems in treating patients with solid tumours: the assessment of the state of the disease; the lack of new effective systemic therapies and the poor selectivity of current agents. Recent advances in molecular genetics have uncovered new targets for therapeutic development. These include growth factors, growth factor receptors, membrane transduction systems, cytoplasmic signal processes and transcription control. The elucidation of the function of dominantly acting oncogenes and tumour suppressor genes provide a key to a new era of molecular-based pharmacological development.

There are several possibilities for the gene therapy of cancer. The first clinical experiment has already been performed involving the insertion of active tumour necrosis factor genes into tumour infiltrating lymphocytes from a patient with melanoma. Such systems all rely heavily on tumour infiltration by appropriately manipulated cells. A more sophisticated gene therapy system involves the insertion of deleterious genes into vectors which can only be expressed in the tumour or cells of the same tissue of origin when not essential for the body as a whole. CEA, AFP, amylase, calcitonin and prostate specific antigen provide examples of such selectively expressed proteins. Retroviral shuttle vectors containing the relevant tumour or tissue specific promoter linked to a potentially toxic product can be constructed in such a way as to only be expressed when inserted into cells which have the necessary transcriptional machinery.

The manipulation of the somatic cell genetics of cancer cells *in vivo* is becoming possible by the use of informational drugs which can downregulate the expression of specific genes. It may also be possible in future to replace defective tumour suppressor genes which may reverse at least part of the malignant phenotype.

The following companies and associations have been major contributors to this meeting: Amgen Roche, Boehringer Ingelheim (Hospital division), Boehringer Mannheim UK Pharmaceuticals, Bookshow Ltd, Bristol-Myers Squibb, Ciba Geigy Pharmaceuticals, Degussa Pharmaceuticals Ltd, Eurocetus UK Ltd, Farmitalia Carlo Erba Ltd, Lederle Laboratories, Merrell Dow Pharmaceuticals Ltd, Nucleotron Trading Ltd, Schering Plough Ltd, Smithkline Beecham, Wisepress Ltd.

### The role of heparin binding growth factors in the malignant progression of human breast cancer

M.E. Lippman, A. Wellstein, F. Kern, S. McLeskey & M. Gottardis

*Lombardi Cancer Research Center, Washington, DC 20007, USA.*

Our laboratory has been investigating the role of secreted growth factor activities in autocrine and paracrine progression of human breast cancer. A survey of tumorigenic human breast cancer cell lines reveals that all secrete heparin binding growth factors capable of inducing clonogenic growth of SW-13 human adrenal carcinoma cells dependent upon fibroblast growth factors for their growth. A variety of known members of the fibroblast growth factor family contribute to these activities as well as yet unidentified activities. We have recently purified, sequenced and cloned a novel heparin binding growth factor produced by some human breast cancer cells which has potent angiogenic potential. In addition, transfection of an expression vector for fibroblast growth factors into human breast cancer cells converts them from hormone dependent to hormone independent growth in athymic nude mice as well as permitting them to form hematogenous metastases. A series of strategies based on binding of heparin binding growth factors to polysaccharides and inhibition of ligand interaction with receptor have been developed which show substantial *in vitro* and *in vivo* antitumour and chemoprevention activities with encouraging therapeutic indices. One of these has been brought into clinical trial for patients with advanced cancer. In summary, heparin binding growth factors appear to play a critical role in both autocrine growth of some breast cancers as well as autocrine promotion of angiogenesis. Interference with these pathways is associated with substantial antitumour activity and is an interesting potential target for breast cancer therapy.

### Exploiting the *ras* oncogene

N.R. Lemoine

*ICRF Oncology Group, Cyclotron Building, Hammersmith Hospital, Du Cane Road, London W12 0HS, UK.*

The three members of the *ras* oncogene family are activated by specific point mutations in a wide range of human tumours. The frequency of activation and the identity of the *ras* oncogene affected vary dramatically between tumour types: 75% of pancreatic cancers have mutations affecting only Ki-*ras* codon 12 and 50% of thyroid tumours have mutations affecting all three oncogenes at codon 12, 13 or 61, while breast cancers and female genital tract tumours do not have *ras* mutations. Activation of *ras* oncogenes often represent an early critical event in tumorigenesis, and has a powerful dominant transforming effect on epithelial cells *in vitro*. Identification of *ras* mutations may be a useful marker for diagnosis of some malignant disease, particularly in situations where only cytological material is available. The presence of *ras* mutations may be a marker of poor prognosis at least in non-small cell lung cancer. New understanding of the mechanisms involved in the processing and control of *ras* proteins and of their connections in signal transduction pathways has identified potential targets for therapeutic intervention. These include interference with synthesis and post-transcriptional modifications required for membrane localisation, interference with the GTP/GDP exchange and GTP hydrolysis, blocking of effector molecule function and exploitation of mutant *ras* peptides as targets for immune therapy.

### p21ras: from onco-protein to signal transducer

J.L. Bos[1], R.H. Medema[1], B.M.Th. Burgering[1], A.M.M. de Vries-Smits[1] & G.J. Pronk[1]

*[1]Laboratory for Physiological Chemistry, Univesity of Utrecht, Utrecht, The Netherlands.*

p21ras proteins are small G-proteins which cycle between a GDP-bound inactive conformation and a GTP-bound active conformation. The proteins play an important role in signal transduction, but the details are still elusive. Genes encoding p21ras proteins are frequently mutated in human tumors. These mutations abolish the intrinsic GTPase activity of the encoded protein, relating in the malfunctioning of p21ras. Our aim is to investigate the consequences of this malfunctioning for the control of cell proliferation. We have chosen fibroblasts as a model system and we have investigated in which growth factor signal transduction pathway p21ras is involved.

Previously, we have suggeted a possible involvement of p21ras in the insulin signal transduction pathway in rat fibroblasts. Recently, we obtained firm evidence for this hypothesis by measuring the effect of insulin stimulation on the activation of p21ras in NIH/3T3 cells overexpressing the insulin receptor. A conversion of rasGDP into rasGTP (20 to 70% GTP as percentage of total nucleotides bound) was found within 1 min after insulin stimulation. Using cells overexpressing high levels of EGF- or PDGF-receptors, we were not able to detect a significant shift in the GTP/GDP ratio after stimulation with EGF or PDGF, stressing the specificity of insulin in the activation of p21ras (Burgering *et al*., EMBO J. 10, 1991, 1103). Using dominant inhibitory mutants of p21ras we have investigated which cellular effect of insulin stimulation is mediated by p21ras. We found that these mutants inhibit insulin-induced activation of the c-fos promoter (Medema *et al*., Mol. Cell. Biol, in press). We conclude from our results that p21ras is involved in insulin-induced signal transduction, in particular in the induction of gene expression. Our results do not exclude a role of p21ras in other signal transduction pathways. Two possible mechanisms of insulin-induced activation of p21ras exist. First, insulin could activate an exchange factor, which substitutes GTP for GDP. Secondly, insulin could inhibit the GTPase activating proteins p120GAP and NF1. An indication that P120GAP may mediate insulin-induced activation of p21ras was obtained using the putative phosphatase inhibitor phenylarsine oxide. This compound revealed an insulin-induced phosphorylation of p120GAP on tyrosines. We, therefore, propose a model in which p120GAP mediates insulin-induced activation of p21ras.

### *myc*, *max* and a novel *rlf*-L-*myc* fusion protein in small-cell lung cancer

K. Alitalo, T.P. Mäkelä, P.J. Koskinen, I. Västrik, G. Evan[1], M.G. Borrello[2], M. Shiraishi[3] & T. Sekiya

*Laboratory of Cancer Biology, University of Helsinki, [1]Imperial Cancer Research Fund, London; [2]Istituto Nazionale dei Tumori, Milan; [3]National Cancer Center Research Institute, Tokyo.*

Max is a recently characterized basic/helix-loop-helix/leucine zipper protein, which forms a sequence-specific DNA-binding complex with the Myc family proteins (Blackwood and Eisenman, *Science* 251, 1211, 1991). We have found a novel Max cDNA which contains the sequences encoding the DNA-binding and dimerization motifs, but lacks both the putative regulatory domain and the nuclear translocation signal of Max. ΔMax retains the ability to form a sequence-specific DNA-binding complex with Myc, but is exclusively cytoplasmic in the absence of Myc. When tested in vivo in a rat embryo fibroblast Myc/ras cotransformation assay ΔMax enhances transformation whereas Max causes a decrease in the number of transformed foci. We suggest that the Max gene encodes both a negative (Max) and a positive (ΔMax) regulator of Myc function.

Oncogenic activation of *myc* genes in human cancer involves deregulated expression of *myc*. Oncogenes of the *myc* family are activated in several types of human tumors as a result of gene amplification or chromosomal translocation. We have recently characterized a gene fusion and a chimeric protein product formed by L-*myc* and a part of a novel gene named *rlf* in two small-cell lung cancer (SCLC) cell lines. Although the chimeric mRNAs were shown to be identical, they result from distinct DNA rearrangements at 1p32. Similar *in vivo* rearrangement involving *rlf* and L-*myc* have been found in a number of cell lines and a primary SCLC tumor. In addition, we have found coamplification of L-*myc* and *rlf* without visible rearrangements in either gene in other SCLC tumors.

### Epidermal growth factor receptor in bladder and breast cancer: relation to p53 expression and target for therapy

A.L. Harris[1], E. Horak[2], S. LeJeune[1], D. Neal[3], K. Smith[1], R. Leek[1], K. Mellon[3] & C. Wright[4]

*[1]ICRF Institute of Molecular Medicine, John Radcliffe Hospital, Oxford; [2]Department of Histopathology, John Radcliffe Hospital, Oxford; [3]Department of Urology, Freeman Hospital, Newcastle upon Tyne; [4]Department of Pathology, Royal Victoria Infirmary, Newcastle upon Tyne.*

Expression of EGF receptor was assessed in 111 primary breast cancers by ligand binding, and compared with p53 expression detected by the antibody PAB240. There was a significant association of p53 expression with EGF receptor expression and inverse relationship with oestrogen receptor expression and p53. There was no association with lymph node status.

A similar study on 82 patients with primary transitional cell cancer of the bladder using immunochemistry showed that 18% of the tumours were positive with p53 with strong staining and a further 36% with weaker staining, giving 54% positive tumours. 31% showed staining for the EGF receptor and 15% for cerbB-2. There was a strong association of tumours invading the bladder muscle with p53 and EGFr staining. However, there was no association between p53 and EGF receptor expression.

EGF receptors were assessed on relapsed and recurrent bladder cancers, approximately half the tumours were positive. Using the toxin TGF alpha PE40, high affinity binding sites were demonstrated with a slightly lower affinity compared to EGF. This toxin was also inhibitory to breast cancer cell lines proportional to the level of expression of EGF receptor. These results suggest that a sub-group of invasive bladder tumours could be treated by intravesical cycle therapy with TGF alpha PE40. Similarly, the high expression of p53 in poor prognosis breast cancer and bladder cancer suggests that approaches involving immunological therapy should be evaluated. To assess whether this would be feasible, a study of the loss of HLA in p53 positive tumours was undertaken in breast cancers. Approximately one third of the patients with HLA A2 showed loss of specific class one HLA molecules. However, the majority of patients still showed the expression of this class one molecule, and therefore would be candidates for immunotherapy.

### Biochemical and clinical applications of monoclonal antibodies against the EGF receptor

J. Mendelsohn

*Memorial Sloan-Kettering Cancer Center and Cornell University Medical College, New York, NY 10021, USA*

We have produced anti-EGFR monoclonal antibodies (MAbs) which block the binding of EGF and TGF-alpha, and can prevent ligand-stimulated activation of EGFR tyrosine kinase. These MAbs have been useful in studies of EGFR function. Experiments utilizing the MAbs to block ligand binding have demonstrated that autocrine stimulation of EGFR phosphorylation can occur via an extracellular pathway, involving TGF-alpha-mediated activation of EGFR on the surface of the cell. The capacity of anti-EGFR MAbs to inhibit cell proliferation has provided evidence for an autocrine stimulatory pathway in cultures of malignant human skin, breast, colon, lung, prostate and kidney cells. Growth of human tumor xenografts can be inhibited in situations where autocrine dependency is demonstrable in cell culture. Imaging studies with anti-EGFR MAb labeled with $^{111}$ Indium demonstrated selective uptake in xenografts expressing high receptor levels. Based on these observations, a phase I trial was carried out with $^{111}$ In-labeled anti-EGFR MAb 225 IgGl in patients with advanced squamous cell lung carcinoma, a tumor which invariably expresses large numbers of EGFR. Escalating doses of 225 IgGl were administered as a single intravenous injection over one hour. A dose of 120 mg 225 IgGl could produce saturating antibody levels in the serum for $>3$ days and successfully imaged primary tumors as well as metastases $>1$ cm in diameter, without producing toxicity. The tumor uptake determined by area-of-interest scanning was 3.4% of the injected dose, and there was considerable uptake in the liver. This study establishes the principle that anti-EGFR agents which block receptor function can be safely administered to patients and will localize, preferentially, to tumor cells bearing high levels of EGFR. Further studies are exploring the potential efficacy of repeated doses of anti-EGFR MAbs, combinations of antireceptor MAbs, and combined MAb plus chemotherapy.

### 1991 Gordon Hamilton-Fairley Memorial Lecture

### Oncogenes and tumor suppressor genes

R.A. Weinberg

*Whitehead Institute for Biomedical Research, 9 Cambridge Center, Cambridge, MA 02142, USA.*

Oncogenes have been studied intensively over the past decade as important determinants of tumor outgrowth. In large part, these genes encode proteins that participate in various ways in the cell's mitogen-response pathway. The various oncoproteins appear to release mitogenic signals constitutively, and in so doing render the cell independent from normally required mitogen stimulation.

Tumor cells must also acquire a second type of growth deregulation, in that they must become non-responsive to growth-inhibitor signals that are present in their environment. Several mechanisms may intervene here. Thus, they may shed receptors for anti-mitogenic signals. Alternatively, they may lose signal-transducing intracellular proteins. The examples of the TGF-$\beta$ receptors and the retinoblastoma gene product will be explored in some detail. Products of tumor suppressor genes confer responsiveness to anti-mitogenic signals and the inactivation of these genes is often critical for tumor outgrowth.

### Transcription factors in growth control and oncogenesis – an overview

H.C. Hurst

*ICRF Oncology Group, Cyclotron Building, Hammersmith Hospital, Du Cane Road, London W12 0HS, UK.*

Cellular growth and differentiation are regulated by numerous proteins whose role is to transduce signals, received at the membrane, across the cell to the nucleus. The proteins that mediate these signals within the nucleus are transcription factors whose role is to bind specifically to the promoter regions of their cognate genes and influence the rate of expression of that gene. Both positively and negatively acting factors are known and indeed some factors may have both activities in different situations. Our current knowledge about how transcription factor activity may be regulated will be reviewed. This includes information on how transcription factors in general are controlled and also how oncogenic versions of these proteins escape this regulation thus contributing to uncontrolled patterns of growth during oncogenesis. Finally, recent data from studies on chromosomal breakpoints associated with human leukaemias will be discussed. This has revealed that novel proteins representing chimeras of two different transcription factors can result from these translocations and the presence of these proteins contributes fundamentally to the transformed state.

### Regulation of cell growth, differentiation and death by *myc*

G. Evan

*Imperial Cancer Research Fund, London.*

Abstract not received

### A genetic screen for SRF/SRE binding proteins

S. Dalton & R. Treisman

*Transcription Laboratory, Imperial Cancer Research Fund, Lincoln's Inn Fields, London, WC2A 3PX, UK.*

Serum Response Factor (SRF) is a 67 kD phosphoprotein that binds to the Serum Response Element (SRE) found in the promoters of the c-fos and several other immediate-early genes (1). Recent work indicates that SRF acts to recruit to the SRE an accessory protein, p62/TCF; although p62/TCF alone does not bind the SRE, in the ternary complex it makes DNA contacts at the SRE left side (2). In Balb/C3T3 cells p62/TCF binding appears to link the c-fos SRE to the protein kinase C dependent signal transduction pathway (3).

A genetic screen using yeast for the isolation of mammalian cDNAs that encode protein that can interact with

SRF at the c-fos SRE will be presented. A reporter strain was constructed that contains a chromosomal lacZ indicator gene placed under control of a UAS consisting of a c-fos SRE derivative. Transformation of this strain with plasmids encoding SRF showed that full length SRF activated the indicator only 4-fold in the presence of galactose, while an SRF-VP16 fusion showed a galactose-dependent induction of more than 100-fold. A library of randomly primed cDNAs from HeLa cell mRNA was constructed in a vector that expresses the cDNAs as fusions with the potent acidic transcription activation domain of the HSV VP16 protein. The library was transformed into the indicator strain and colonies that activated the reporter gene identified by β-galactosidase color assay. cDNAs were recovered that activated the reporter gene in both an SRF-dependent and -independent fashion. The structure and properties of the encoded proteins will be discussed.

**References**

TREISMAN, R. (1990) Seminars in Cancer Biology, **1**, 47.
SHAW, P. & others (1989) *Cell*, **56**, 563.
GRAHAM, R. & GILMAN, M.Z. (1991) *Science*, **251**, 189.

### Constance Wood Memorial Lecture

### Retinoid receptors in development and disease

R.M. Evans*[1], A. Kakizuka, S. Kliewer, D. Mangelsdorf & K. Umesono[1]

*Howard Hughes Medical Institute[1], The Salk Institute, La Jolla, CA 92037.*

The cellular responses to RA are mediated by two families of transcription factors, which include the RA receptors (RARs) and the retinoid X receptors (RXRs). Although both RAR and RXR respond specifically to RA, they differ substantially from one another in primary structure and ligand specificity. A major question raised by the discovery of two retinoid-responsive systems is whether their functions are independent, interactive, or redundant. One approach to answer this question is to determine whether they share common or distinct downstream target genes. In regard to target sequences we have recently described properties of direct repeats (DRs) of the half-site AGGTCA as hormone response elements. According to our results, spacing of the half-site by 3, 4, or 5 nucleotides determines specificity of response for vitamin D3, thyroid hormone and retinoic acid receptors, respectively. This so-called '3-4-5' rule suggests a simple physiologic code exists in which half-site spacing plays a critical role in achieving selective hormonal response. As part of these studies, we have also identified that the RXR, but not the RAR, is able to activate through a direct repeat spaced by one nucleotide. In contrast, both RAR and RXR are able to commonly activate through a DR with a spacing of 5. Evidence that RXR heterodimers modulate the RA response will be presented. Finally we will discuss the isolation and characterization of a fusion product produced as a consequence of a t(15:17) translocation characteristic of human acute promyelocytic leukemia. This translocation which occurs in the retinoic acid receptor gene generates a unique mRNA which encodes a fusion protein between the retinoic acid receptor alpha (RARα) and a myeloid gene product called PML. Structural analysis reveals that the PML protein is a member of newly recognized protein family that includes a variety of putative transcription factors as well as the recombination-activating gene product (RAG-1). The abberant PML-RAR fusion product, while typically retinoic acid responsive, displays both cell type and promoter specific differences from the wild type RARα. Because patients with APL can be induced into remission with high dose RA therapy, we propose that the non-liganded PML-RAR is a new class of dominant negative oncogene product.

### Abstracts of members' proffered papers

### The importance of serum concentrations on the efficacy and toxicity of suramin in patients with advanced cancer

J. Wagstaff[1], R.E.N. van Rijswijk[1], A.C. van Loenen[2], R. Lopez[1], C.J. van Groeningen[1], J.J. Heimans[3] & H.M. Pinedo[1]

*[1]Department Medical Oncology, [2]Department Pharmacy, [3]Department Neurology, Free University Hospital, 1007 MB Amsterdam, The Netherlands.*

Aberrations in growth factor activity have been clearly implicated in the promotion of cancer cell proliferation. Suramin (Sur) has the ability to interfere with these processes *in vitro* and consequently the drug has been explored as a new anticancer agent in the clinic. Sur levels of >200 mg/L have generally been required before anti-tumour responses have been observed whilst levels >300 mg/L have been associated with disabling neurotoxicity. This narrow therapeutic window has meant that the control of serum Sur levels has become a crucial issue in the clinical development of this drug. The terminal plasma half life of Sur is 45–55 days which makes a loading regimen designed to achieve therapeutic serum levels (200–300 mg/L) followed by intermittent maintenance treatments rational.

We have examined the pharmacokinetics of 5 loading regimens in an attempt to discover the optimum schedule for sur. 1–1.5 gm/week for 3 to 6 weeks produced levels >200 mg/L in only one patient (pt) whilst 3/6 developed progressive disease before this level was obtained. 350 mg/m$^2$/day, as a continuous infusion (4 pts) produced levels of 160–247 mg/L after 6 to 14 days whilst 600 mg/m$^2$/day (4 pts) gave levels of 264–381 in 3–5 days. This latter dose if given by 6 (13 pts) or 12 (5 pts) hour infusion may give peak levels in excess of 400 mg/L after 3 to 5 days.

The weekly maintenance treatments were based on the trough Sur levels and varied from 300 to 900 mg/m$^2$. There was a linear relationship between these doses and the mean increase produced. A regression line drawn through these mean values has an r value of 0.999 (<0.0001). Using these data and knowing the trough and target (≅300 mg/L) Sur levels it is possible to calculate how much Sur to give. Using a continuous infusion loading regimen of a few days duration and the above data to calculate the maintenance doses it is possible to maintain the Sur levels within the narrow therapeutic window. Studies conducted until now have failed to adequately achieve this. Further studies are indicated in order to determine the efficacy and toxicity of Sur when the drug levels are maintained within this narrow therapeutic window.

### Sequential interleukin 2 and alpha interferon for renal cell cancer and melanoma

H. Thomas, C. Barton, A. Saini, A. Dalgleish & J. Waxman

*Department of Clinical Oncology, Royal Postgraduate Medical School, Hammersmith Hospital, Du Cane Road, London W12 0NN, UK.*

Unexpectedly high response rates to Interleukin 2 therapy have been described in renal cell carcinoma and melanoma.

The consensus view is that the true response rate is lower than initially reported and its toxicity, greater. There is a theoretical basis for the synergy of interleukin 2 with other cytokines. We have investigated sequential treatment with interleukin 2 and alpha interferon. Thirty one patients with metastatic renal cell carcinoma or melanoma have been treated with continuous infusion recombinant interleukin 2 (Glaxo). One of twenty two patients had a partial response and one a minimal response. None of nine patients with melanoma responded to interleukin 2. Sixteen of seventeen patients with renal cell cancer and eight with melanoma subsequently received alpha interferon (Roche). Two patients with renal cell cancer responded to alpha interferon with sustained remissions of two and three years; both had responded to interleukin 2. The further investigation of cytokine combination therapy is suggested by these unusually long responses to alpha interferon.

### Cytogenetics of uveal melanoma

K. Sisley[1], D. Cottam[2], G. Rennie[2], A.M. Potter[3], C.W. Potter[1] & R.C. Rees[1]

*[1]Department ECM and [2]Department Opthalmology, Medical School, Sheffield University; [3]Centre for Human Genetics, 117 Manchester Road, Sheffield.*

Posterior uveal melanoma is an ocular tumour derived from the ciliary body and choroid. We have studied 20 of these ocular tumours of which 16 were successfully analysed. Cytogenetic reports of this tumour have been limited, with only 23 reported cases. We present the results from 16 tumours. Two tumours had a normal chromosome complement, and a second demonstrated loss of the Y chromosome as the single abnormality. The remaining tumours presented basically pseudodiploid karyotypes with only limited chromosome changes. The most frequently involved chromosomes were 3, 6 and 8. Seven tumours derived from the ciliary body had non random association between monosomy of chromosomes 3 and i(8q). Abnormalities of chromosome 6 involving the long or short arms were found in 9 tumours and two tumours also demonstrated deletion at 11q23.

### Loss of heterozygosity (LOH) at the 11p15 locus in human ovarian cancer.

D. Eccles[1], L. Gruber[2], M. Steel[2] & R. Leonard[1]

*[1]Imperial Cancer Research Fund, Medical Oncology Unit, [2]MRC Human Genetics Unit, Western General Hospital, Edinburgh EH4 2XU.*

Detection of areas of the human genome where allele loss is a common feature of a particular type of tumour is a popular method of identifying candidate tumour suppressor genes (tsg). The hallmark of a tsg is inactivation of the gene at both loci. LOH is frequently identified as one inactivating event and once the tsg has been isolated and cloned, mutations are usually identifiable in the retained copy. We have examined 46 ovarian tumours with paired blood DNA samples for LOH at two loci in the 11p15 region, the c-Ha-*ras* (11p15.5) locus and the more proximal calcitonin (CALC) locus (11p15.5). Out of 24 serous carcinomas allele loss at 11p15.5 was only 14% (2/14 informative tumours) compared with 46% (5/11) at the CALC locus. Out of all types of carcinoma, LOH at CALC was still significant at 33% compared with the rate at the c-Ha-*ras* locus of 18% overall. There may be a tsg at or near the CALC locus which is implicated in the genesis of ovarian carcinoma. One out of 2 informative borderline tumours showed allele loss at CALC but retained heterozygosity at the distal locus. The molecular events involved in the development of borderline tumours may be a step on the way to the development of frank carcinoma.

### Phosphomonoester and proliferation in human breast cancer

R. Kalra, K. Wade[1], M. Greenall[2], R. Camplejohn[3], A.L. Harris[4] & G.K. Radda[1]

**MRC Radiobiology Unit, Chilton; [1]Biochemical and Clinical Magnetic Resonance Unit, [2]Department Surgery and [4]Clinical Oncology, John Radcliffe Hospital, Oxford; and [3]R. Dimbleby Department Cancer Research, St Thomas' Hospital.*

$^{31}P$ magnetic resonance spectroscopy (MRS) of tumours show elevated levels of phosphomonoester (PME). The PME region contains predominately the synthetic precursors of membrane phospholipids, phosphoethanolamine (PE) and phosphocholine (PC). Early changes in PME following therapy of breast cancer have been demonstrated. In cultured human breast cancer cells the levels of PE and PC are greater during exponential growth than at confluence suggesting an association between the PME content and proliferation.

PME/ATP was measured from localised phosphorus spectra in 31 patients with early breast cancer undergoing surgery. PE and PC concentration was calculated from high resolution $^{31}P$ spectra of tumour extracts obtained at surgery including additional cases with poor *in vivo* spectral resolution. Tumour tissue was also taken for histology, and DNA flow cytometry for calculation of S phase fraction (SPF) for assessment of tumour proliferation. Mean PME/ATP was 1.65 (sd $\pm$ 0.86) with a greater concentration of PE than PC. The mean SPF in 27 cases was 8.36% (sd $\pm$ 7.49%). There was no correlation between PME/ATP, PE, PC and SPF or histological grade.

The relative amounts of PE and PC depend not only on proliferation rate but are also affected by the conditions of growth and the cell line suggesting genetic differences.

### Evaluation of cell kinetics of cervical carcinoma

B.S. Bolger[1], T.G. Cooke[2], P.D. Stanton[2], A.B. McLean[1] & R.P. Symonds[3]

*[1]Department of Midwifery, Queen Mothers Hospital, Glasgow; [2]Department of Surgery, Glasgow Royal Infirmary; [3]Department of Oncology, Western Infirmary, Glasgow.*

Tumour cells kinetics may indicate prognosis and help to predict response to chemo- or radio-therapy. We have sought to establish the cell kinetics of cervical carcinomas, using the newly available technique of in vivo bromodeoxyuridine labelling.

Sixty patients with stages Ib-IVb cervical carcinoma were given bromodeoxyuridine 6–8 h prior to tumour biopsy. Disaggregated fixed nuclei were stained for bromodeoyxuridine and total DNA content, and analysed by flow cytometry. The proportion of bromodeoxyuridine labelled nuclei (the labelling index, LI), the length of S phase (Ts), and the potential doubling time (Tpot) were then derived.

Fifty two (87%) of tumours produced satisfactory results. Median parameters were: LI 7.5% (range 1.1–22.5), Ts 13.0 h (range 7.4–21.5), Tpot 6.6 days (range 1.5–52.5). Advanced tumours had significantly higher LI than early stage lesions (LI median 8.4% vs 3.9%, $p = 0.022$, Mann Whitney). Normal cervical tissue was obtained from 8 patients, and showed significantly lower LI (0.8%, $p < 0.001$), but no difference in Ts.

These preliminary results demonstrate the feasibility of measuring cell kinetic parameters in patients on a routine basis. Bromodeoxyuridine labelling index is associated with tumour stage, but the length of S phase shows no variation with stage or presence of tumour.

**Oestrone sulphatase inhibitors as antitumour agents for oestrogen-dependent breast cancer**

A. Purohit, L. Duncan & M.J. Reed

*Unit of Metabolic Medicine, St Mary's Hospital Medical School, London W2 1PG, UK.*

Approximately one third of human breast cancers are oestrogen dependent. Such tumours regress upon endocrine treatment. Oestrone sulphate stimulates growth of breast tumours via conversion to oestrone and oestradiol as shown by data from a variety of *in vitro* and *in vivo* studies. Oestrone sulphatase, the enzyme which converts oestrone sulphate into oestrone, is consistently found in primary mammary carcinoma. This oestrone sulphatase pathway may, therefore, be significant and perhaps the primary means of local production of oestrone in breast tissues. We have synthesised and evaluated a steroidal inhibitor of oestrone sulphatase.

Oestrone-3-methylphosphonothioate ($E_1$MPT) was found to competitively inhibit *in vitro* oestrone sulphatase activity in both intact breast cancer cells and human placental tissue. The Km for oestrone sulphate was 6.13 μM whereas the Ki for $E_1$MPT was 14.56 μM in placental tissue. The $IC_{50}$ values when oestrone sulphate was used at a concentration of 10 μM were: 43 μM (placenta) and 36 μM (breast tumour). The development of inhibitors of oestrone sulphatase represents a novel therapeutic approach to hormone-dependent breast cancer.

**Glycoprotein processing inhibitors may inhibit tumour metastasis by potentiating anti-tumour immune responses**

C. Galustian, S. Foulds, J.F. Dye & P.J. Guillou

*Academic Surgical Unit, St Mary's Hospital Medical School, London W2 1NY.*

Swainsonine (SW) inhibits Mannosidase II during N-linked glycoprotein processing. SW has recently been found to inhibit metastasis in several murine tumour models and to augment the expression of receptors for the lymphokine Interleukin-2(IL-2) on lymphocytes. In this study we have asked whether SW might achieve these antimetastatic effects by potentiating anti-tumour immune responses. The effect of SW on the generation of lymphokine-activated killer (LAK) cells was examined by performing SW dose response curves on the generation of LAK cells from human peripheral blood mononuclear (PMN) cells in response to a fixed concentration of IL-2 (1000 units/ml) in vitro for 3 days. Data are expressed as mean Area-under curve (AUC) units at each concentration of SW. At a concentration of 2 μg/ml, SW significantly augmented LAK activity from 138 AUC units to 166 (± SED 10) AUC units ($P<0.05$ by Student's t-test) and was dose-dependant ($P<0.05$ by ANOVA, n = 6 experiments).

We also studied the effects of SW on the susceptibility of tumour cells to killing by human LAK cells. COLO320 colorectal cancer cells were cultured for 3 days with varying concentrations of SW before being used as target cells in the LAK cell assay which used unmodified PMN activated for 3 days with 1000 units/ml IL-2 alone as the effector cells. The results for these experiments were as follows:- (mean of 3 experiments)

| Concn. SW (μg/ml used to modify COLO320 | 0 | 1.0 | 2.0 | 5.0 | 10.0 |
|---|---|---|---|---|---|
| LAK(AUC units ± SED) | 75 | 93 ± 6.9 | 100 ± 5** | 94 ± 1.5** | 90 ± 3.3 |

(**$P<0.05$ on comparison with control by paired t-test).

Thus SW significantly augments human LAK activity and tumour cell susceptibility to LAK cells at a concentration of 2 μg/ml. SW is non-toxic and may be an important reagent for increasing the response rates of patients with malignant disease to IL-2 therapy.

**Activated polymorphonuclear neutrophils - mediators of the interleukin-2 induced capillary leak syndrome?**

C.H. Wakefield, P.D. Carey, S. Foulds, J. Monson & P.J. Guillou

*Academic Surgical Unit, St. Mary's Hospital Medical School, London W2 1NY.*

Interleukin-2 (IL-2)-based therapies provide both the greatest response rates and prolongation of survival in patients with advanced malignant melanoma (MM) and renal cell cancer (RCC). Response rates are related to the total IL-2 dose administered but the principal dose-limiting toxicity is a capillary leak syndrome (CLS) of obscure aetiology which predominantly afflicts the lung. Severe sepsis is also associated with a similar acute lung injury which is mediated at least in part by activated neutrophils. Tumour necrosis factor (TNF) is a potent activator of neutrophils and may be released during IL-2 infusion. We sought evidence for neutrophil activation in 10 patients with advanced MM or RCC who developed CLS during a 5-day therapeutic infusion of IL-2. Plasma TNFa levels were measured before (baseline) and on days 1 and 5 of the infusion. Simultaneously, the expression of the neutrophil activation marker CD11b and neutrophil production of $H_2O_2$ were measured by flow cytometry and are expressed as the mean (± SEM) of the mean channel fluorescence (MCF) for each parameter.

| | Plasma TNFα (pg/ml) | $H_2O_2$ PRODN (MCF) | CD11b expression (MCF) |
|---|---|---|---|
| Baseline | 7.8 ± 1.3 | 103.2 ± 4.5 | 75.4 ± 9.8 |
| Day 1 | 53.9 ± 10.1** | 111.9 ± 3.7** | 121.1 ± 18.9** |
| Day 5 | 28.3 ± 9.6 | 111.1 ± 4.4** | 197.6 ± 34.1** |

**$p<0.05$ by paired t-test on comparison with baseline data.

Plasma TNFα levels are elevated within 24 hours of beginning IL-2 infusion and this is accompanied by phenotypic and functional neutrophil activation. This is strong evidence of a role for activated neutrophils in IL-2-induced CLS and suggests means for modifying IL-2 toxicity without necessarily diminishing its therapeutic efficacy.

**Localisation of BHRFI to the mitochondria by immunoelectron microscopy: implication for a role in preventing cell death by apoptosis**

T. Hickish[1], C. Clarke[2], D. Robertson[2] & D. Cunningham[1]

[1]*Section of Medicine,* [2]*Section of Pathology; Institute of Cancer Research, Royal Marsden Hospital, Sutton, Surrey.*

BHRFI is an Epstein-Barr virus gene of unknown function which is expressed at the interface between the latent and lytic cycles[1]. Cell fractionation studies have indicated it is associated with the mitochondria. BHRFI has 40% homology with the bcl2 gene[2] which is deregulated in at least 90% of cases of follicular non-Hodgkin's lymphoma. Bcl2 is expressed at the inner mitochondrial membrane and deregulation appears to extend cell survival by interrupting cell death by apoptosis[3]. BHRFI and bcl2 therefore may have functional equivalence.

To explore the function of BHRFI we have examined an EBV-genome positive cell line, B95.8, using low temperature embedding immunoelectron microscopy. SU-DHL4, a cell line which expresses bcl2 was also studied. B95.8 cells in log phase growth were cultured in either standard (10% foetal calf serum) or stressed (foetal calf serum absent) conditions for three days. SU-DHL4 cells in log phase growth were cultured in standard conditions. Cells were then pelleted, washed, embedded in resin and 0.1 μ sections prepared for electron microscopy. Expression of BHRFI and bcl2 were determined using the 5B11 and 100 antibody respectively.

| | Doubling time | % Viability at 3 days | BHRFI expression | bcl2 expression |
|---|---|---|---|---|
| B95.8 Standard | 24 hrs | >95% | 0.6% cells | 0% |
| B95.8 Stressed | 30 hrs | >95% | 3.5% cells | NT |
| SU·DHL4 | 24 hrs | >95% | 0% | >90% |

B95.8 cells undergoing apoptosis could be identified by their distictive nuclear chromatin pattern[4] and these cells contained clearly visible virions and were therefore in the lytic cycle. The proportion of cells in the lytic cycle was increased in the stressed B95.8 cells. Only B95.8 cells in the lytic cycle expressed BHRFI and this was displayed at the mitochondria. Mitochondria of lytic cycle B95.8 cells did not cross react with 100.

One possible interpretation of this data would be that BHRFI is expressed by EBV as it enters the lytic cycle to extend the lytic cells' survival thus enabling maximum virus production.

There is emerging detail of a pathogenic role for EBV in Hodgkin's disease[5,6]. Currently we are exploring the possibility that BHRFI may promote Reed Sternberg cell survival.

This is the first demonstration of BHRFI expression at the mitochondria and is an example of the utility of immunoelectron microscopy for investigating gene function.

(5B11 was kindly provided by Dr G. Pearson and 100 was a gift from Dr D. Mason).

#### References

PEARSON, G. & others (1987). Virology. **160**, p151.
KOACHE, M. & others (1990). Intervirology. **31**, p1.
HOCKENBERY, D. & others (1990). Nature. **348**, p334.
HERBST, H. & others (1991). *Proc. Natl Acad. Sci. USA.* **88**, p4766.
PALLESEN, G. & others (1991). *Lancet.* **337**, p320.

### Weekly cisplatinum in combination as salvage for germ cell tumour patients failing standard 3 weekly platinum based regimes

M.A. Raja, R.T.D. Oliver, J. Ong & C.J. Gallagher

*Department of Medical Oncology, The Royal London Hospital, Whitechapel, London E1 1BB.*

Recently there have been reports correlating chances of success with standard 3 weekly platinum based regimes and an index of tumour growth rate (Price - E.J. Cancer 1990; 26: 453) in malignant teratomas. Encouraged by several reports in the literature that it was possible to give Cisplatin more frequently than every three weeks (Merrin - J Urol 1978, 120: 73, Newlands - Lancet 1983; 1: 948, Wetlaufer - Cancer 1984; 53: 209), we have developed a regime alternating weekly BOP with M-BOP.

Twenty-three patients with metastatic malignant teratoma, 4 relapsing after CR and 19 failing to achieve CR with primary chemotherapy (6BEP(5), 8 EBCi (3), 8 EBCa (3), and 1 Carboplatin), have been treated with this regime. Patients received a median of 6 weeks of treatment with a range of 3–10 weeks. 16 patients achieved CR, 11 of these with surgery immediately following chemotherapy for removal of residual masses (8 Retroperitoneal lymph node dissection, and 3 thoracotomy out of which 6 showed foci of mature teratoma, and 5 necrosis only). 11 of these continue in CR with a median follow up of 14 months (range 6–60 months).

### Prognostic significance of mitotic index and c-erbB2 oncogene product in childhood medulloblastoma

R.J. Gilbertson[1], A.D.J. Pearson[1], E.B. Jaros[2] & R.H. Perry

*[1]Department of Child Health, Medical School, Newcastle upon Tyne, NE2 4HH; [2]Department of Neuropathology, Newcastle General Hospital, Newcastle upon Tyne, NE4 6BE.*

The prognostic significance of mitotic index (MI) and c-erbB2 oncogene product expression of cells was investigated in 52 children with medullo-blastoma. The MI was calculated for each case determining the percentage of non-disputable mitotic figures within standard haematoxylin and eosin sections prepared from all available tumour blocks. Expression of the c-erbB2 oncogene product was detected immunohistochemically using the monoclonal antibody NCL-CB11 and the avidin biotin peroxidase complex technique. Univariate analysis demonstrated a significant stepwise decrease in patient survival with increasing MI. The 10 year survival rates for the categories MI 0–2%, 2–3% and >3% were 42%, 33% and 0% respectively ($p < 0.0001$). Forty six (83.6%) tumours were positive for the c-erbB2 protein. All staining was abolished using antigen absorption controls. Patients with more than 50% of tumour cells showing c-erbB2 product positivity had a reduced survival ($p < 0.005$) – only 10% of these patients were alive at 10 years following diagnosis compared to 55% of cases with less than 50% positive cells. MI and c-erbB2 oncogene product expression were analysed further using correlation statistics and multivariate survival analysis. A significant relationship between tumour c-erbB2 protein expression and MI was observed ($p < 0.001$). When analysed together in multivariate analysis, MI retained prognostic significance ($p = 0.004$) whereas c-erbB2 protein expression did not. Tumour MI represents a highly significant independent prognostic factor for childhood medulloblastoma. C-erbB2 oncogene product expression is closely related to tumour MI but does not have independent prognostic significance.

### Abnormalities of the p53 tumour suppressor gene in pancreatic cancer

C.M. Barton[1], S.L. Staddon[1], C.M. Hughes[1], P.A. Hall[2], C. O'Sullivan[1], G. Kloppel[3], B. Theis[4], R.C.G. Russell[4], J. Neoptolemos[5], R.C.N. Williamson[6] & N.R. Lemoine[1]

*[1]ICRF Oncology Group, Hammersmith Hospital, London; [2]Department of Histopathology, Hammersmith Hospital; [3]Department of Pathology, Academic Hospital Jette, Free University of Brussels, Belgium; [4]Department of Surgery, Middlesex Hospital, London; [5]Department of Surgery, Dudley Rd Hospital, Birmingham; [6]Department of Surgery, Hammersmith Hospital, London; [7]Cell Transformation Research Group, CRC Laboratories, Department of Biochemistry, University of Dundee, Dundee.*

The tumour suppressor gene p53 has been found to be mutated or inactivated at high frequency in several common human tumours. Mutant forms of the p53 gene classically cooperate with activated *ras* oncogenes to transform cells *in vitro*. We have examined a series of exocrine pancreatic carcinomas for overexpression of mutant forms of p53 by immunohistochemistry with a panel of specific antibodies. We found immunodetectable p53 in 13 of 22 (60%) frozen pancreatic cancers and 7 of 13 pancreatic cell lines. One of the antibodies, CM1, recognises p53 in formalin-fixed, paraffin-embedded archival material and using this reagent we found immunodetectable p53 in 28 of 124 (23%) pancreatic cancers. We have successfully demonstrated the presence of point mutations by direct sequencing of genomic DNA extracted from archival tissue showing CM1 immunoreactivity. Overexpression of p53 protein was also detectable in 5/13 pancreatic cancer cell lines by immunoprecipitation and mutations in the p53 gene were confirmed by direct sequencing of genomic and complementary DNA in these lines. Pulse chase analysis indicated that the mutant protein has a prolonged half-life. We conclude that p53 activation is an important event in human pancreatic tumourigenesis and that the CM1 antibody is of value for the analysis of archival pathological material.

**Mutation analysis of p53 in human normal, adenoma, diverticular and carcinoma colorectal tissues**

N.J. Froggatt[1], S.H. Leveson[2] & R.C. Garner[1]

*Cancer Research Unit, Department of Biology, University of York, Heslington, YO1 5DD, England; [2]York District Hospital, Wigginton Road, YO3 7HE, England.*

Base substitution mutations in the p53 gene constitute one of the most frequently occurring genetic lesions identified so far in the multi-stage progression of cancer, having been found in tumours originating from diverse tissue types. We have examined paired normal and neoplastic human colorectal tissues (both benign and malignant) for mutations in p53 using the polymerase chain reaction to amplify one region of interest, comprising exons 5, 6, 7, and intervening introns.

Preliminary screening of amplified samples was carried out using three restriction endonucleases, *Cfo I, Hpa II,* and *Hae III*, which cleave in normal tissues at codons 175, 248 and 249, all sites at which mutations have previously been found. Abnormal band patterns were seen in 7 of 20 tumour samples (1 adenoma and 6 carcinomas) as compared with corresponding normal tissues, but not in diverticular samples.

Direct detergent-based sequencing of the PCR products has confirmed these base substitution events, and has also revealed mutations at codon 245, another common mutation site. G.C to A.T transitions at CpG dinuleotides so far predominate; C.G to T.A transitions have also been found.

This work adds significantly to the number of mutations found in human colorectal neoplasms. It confirms the lack of a single codon as a mutational hotspot, but supports the hypothesis that GC-rich sequences are the loci most often involved.

**Implications of alterations in the structure of the p53 tumour suppressor gene in human breast cancer**

D.M. Barnes[1], C.J. Fisher[1], C.E. Gillett[1], R.R. Millis[1] & D.P. Lane[2]

*[1]ICRF Clinical Oncology Unit, Guy's Hospital, London, UK; [2]CRC Labs, University of Dundee, Dundee, UK.*

The p53 gene encodes a 393 amino acid nuclear phosphoprotein which is now thought to act as a tumour suppressor. Alterations in the structure of the gene (usually either allele loss and/or mutation) are common in many human cancers. The wild type protein has a very short half-life and rapidly disappears from the cell. Mutations, which appear to confer stability, lead to abnormal acccumulation of protein which can be detected immunohistochemically.

We have used polyclonal antibody CM-1 (Midgeley *et al.*, manuscript submitted) raised against a construct of the entire p53 protein, in an immunohistochemical study of breast carcinoma. Optimum conditions have been established for the immunohistochemical detection of the protein in formalin fixed paraffin-embedded tissue. Positive nuclear staining, ranging from weak to very strong, is seen in approximately 70% of cases of infiltrating carcinoma. Care must be taken when interpreting lack of positive staining as this could occur either when the p53 gene is unaltered or when both alleles have been lost. Staining patterns have been related to tumour morphology and a significant association has been found between strong staining and poorly differentiated grade III infiltrating ductal carcinomas.

Early work on ductal carcinoma in situ suggests a lower incidence of positive staining than occurs in infiltrating tumours. No relationship has been found, so far, between staining for p53 and the morphology of the in situ tumours.

**Mutations in exon 7 of the p53 tumour suppressor gene are not pathognomonic of Li Fraumeni syndrome**

J.M. Dunn[1], D.J. Hastrich[1], S. Nicholson[1], N.J. Maitland[2] & J.R. Farndon[1]

*[1]Department of Surgery, Bristol Royal Infirmary, Bristol BS2 8HW; [2]Department of Pathology, University of Bristol.*

Abnormalities of the p53 tumour suppressor gene have been detected in many human malignancies. Mutations have been detected in specific regions of the gene and some may have a significant role in malignant transformation and tumour progression. Germline mutations of the p53 gene at codons 245, 248, 252 and 258 have recently been detected in Li Fraumeni syndrome. This syndrome is characterised by the association of childhood sarcoma with an increased incidence of early onset, multiple primary tumours of breast, brain, larynx, bone, adrenal gland and leukaemia among family members.

One family demonstrating characteristics of Li Fraumeni syndrome have been examined at the 17p locus by Southern blot and PCR-SSCP (polymerase chain reaction single strand conformation polymorphism) techniques. The family includes a 16 year old female with glioblastoma, her brother with a sarcoma at 19 years and mother with bilateral breast cancer who had a mastectomy at 32 years.

The index case demonstrates loss of the paternal copy of the 17p allele in brain tumour tissue. Screening the family by PCR-SSCP of exons 5–8 inclusive revealed no germline mutations the p53 gene. PCR sequencing of the index case at exon 7 detected no base changes.

Mutations in exon 7 are not pathognomic of Li Fraumeni syndrome. Loss of the normal paternal 17p allele in this tumour may uncover p53 abnormalities in other exons which merit further investigation.

**Are inherited mutations of the p53 gene a general determinant of familial breast cancer?**

R. Eeles[1,2], W. Warren[2], B. Ponder[4], D. Averill[3], D. Easton[3], M. Ponder[4], C. Cooper[2] & J. Peto[3]

*Departments of Academic Radiotherapy, Royal Marsden Hospital[1], Molecular Carcinogenesis[2], and Epidemiology[3], Institute of Cancer Research, Surrey. CRC Human Genetics Unit, Cambridge[4].*

Recent evidence links the Li-Fraumeni syndrome to inherited mutations within exon 7 of the p53 gene*. Since an excess of breast cancer is found in this syndrome, and somatically acquired mutations of exons 5–9 of the p53 gene are a common feature of sporadic breast cancer, these findings have prompted speculation that germline p53 mutations may be important in familial breast cancer. We have examined lymphocyte DNA from 42 individuals with a strong family history of breast cancer for germline mutations in exons 5–9 of the p53 gene. These individuals were from 26 families and in all families the index case was $\leqslant 46$ years old at diagnosis. In 7 families there were 2 affected first degree relatives aged $<45$ at diagnosis and in 12 families there were 3 affected first degree relatives, 2 of whom were $<45$ at diagnosis. In 15 families, 2 affected members were examined. The PCR amplification products of exons 5, 6, 7, and 8 + 9 were examined by single-stranded conformational polymorphism analysis (SSCP) and for exon 7 the PCR product, synthesised using a biotinylated primer, was also solid phase-sequenced using streptavidin-coated magnetic beads.

No germline mutations were detected by SSCP in exons 5-9 of the p53 gene. In addition, direct sequencing of exon 7 also failed to show any mutations. We therefore conclude that most familial breast cancer is unlikely to result from such mutations.

**References**

MALKIN, D., Li FP & others (1990). *Science*, **250**, 1233-1238.

**Tumour heterogeneity of ras and p53 abnormalities in colorectal carcinoma**

M.I. Ahamed[1], D. Grimshaw[1], G.T. Williams[2], M.C.A Puntis[1], L.E. Hughes[1] & R.A. Padua[3]

*Departments of [1]Surgery, [2]Pathology, [3]Haemotology, University Hospital of Wales, Heath Park, Cardiff CF4 4XN.*

The dynamic heterogeneity of colorectal tumours was examined in terms of oncogene expression in 19 patients at different stages of disease. RAS and p53 oncogenes were studied in normal mucosa, adenomatous polyps, primary tumour and nodal and liver metastases. RAS mutations at codon 12 of KRAS were analysed using polymerase chain reaction followed by hybridization with oligonucleotide probes. Alterations in p53 gene were analysed by enzymatic digestion with two restriction enzymes. Hind III and Xbal, and hybridisation with c DNA probes using the Southern Blot technique. 15 specimens from 6 patients showed point mutation at codon 12 of KRAS, 4/4 villous polyps, 0/2 tubular polyps, 4/18 primary cancers, 2 of 6 positive nodes and 3 of 4 hepatic metastases. Nearly 50% of patients (9/19) showed alteration of p53 gene, either restriction fragment length polymorphism or allele loss in different tissue specimens of 2 polyps, 9 primary tumour and 3 each of nodal and liver metastases. Four patients had both RAS and p53 mutations. While RAS and p53 oncogenes occurred in 32% and 50% of patients respectively, the mutation is not stable throughout the disease process.

**Differential regulation of growth and EGF receptor expression by retinoic acid in prostatic cell lines of normal and neoplastic origin**

T.D. France & C.L. Eaton

*Tenovus Institute for Cancer Research, Heath Park, Cardiff CF4 4XX.*

Although 70% of prostatic cancers respond to androgen ablation as primary therapy, the disease is inevitably refractory to this treatment and the subsequent behaviour of these tumours is indicative of increasing autonomy in respect of growth. Several studies have suggested that EGF and related peptides are important growth regulators in normal and neoplastic prostatic cells. Retinoic acid is an established morphogen and is potentially interactive with the action of growth factors and subsequent signal transduction pathways. We therefore investigated the proliferative effects of retinoic acid and the concomitant influence upon expression of the EGF receptor in prostatic epithelial cell lines.

In serum-free cultures of two poorly differentiated tumour prostatic cell lines (PC3, DU145) retinoic acid was a significant growth enhancer. In contrast, retinoic acid was a growth suppressor in cell lines derived from normal tissue (CAPE) and from a well differentiated tumour (CPA) and abolished the potent growth promoting properties of EGF in these cell lines. EGF receptor expression was measured at the protein ([$^{125}$I]EGF saturation analysis) and mRNA (Northern hybridisation) level. In CPA cells retinoic acid significantly elevated EGF receptor expression, while in cells derived from normal prostate (CAPE) retinoic acid decreased receptor expression. The effects of other agents, including androgens, upon EGF receptor expression were also disparate.

These data suggest that differences in retinoic acid sensitivity and EGF response pathways exist between prostatic cell types of varying pathology.

**Differential expression of oestrogen responsive genes in breast cancer**

D.L. Manning & R.I. Nicholson

*Tenovus Institute for Cancer Research, Heath Park, Cardiff CF4 4XX.*

Our inability to accurately predict by ER status alone whether a given patient will respond to endocrine therapy has led to the search for additional markers of hormone responsiveness.

We have prepared two cDNA libraries from the mRNA of oestrogen stimulated breast cancer cells (ZR-75-1 and T-47D-5). A total of ten genes (pLIV-1, pLIV-2 (pS2) and pSyd 1-8) have been isolated whose mRNAs are increased by oestrogen (2.5-16 fold) and reduced by antioestrogens. In order to determine their potential as prognostic markers in the management of breast cancer we have measured the expression of four genes (pLIV-1, pLIV-2; pSyd 3 and pSyd 8) in 118 primary breast tumours which were also immunohistochemically assayed for ER, PgR, ErbB2, EGFR, Ki67 and pS2. All four genes were expressed at higher levels in tumour compared to those found in normal breast tissue and cell lines. In addition, pSyd3 and 8 sequences were more abundant than their pLIV-1 & 2 counterparts. Both pLIV-1 and -2 showed a strong correlation with ER status with expression increasing with increased cellular ER positivity. An inverse relationship between ER and EGFR levels was observed and was reflected in pLIV-1 and -2 mRNA levels decreasing with increasing cellular EGFR staining. In contrast to pLIV-1 and pLIV-2, pSyd-3 and -8 were expressed in both ER – and ER + tumours and showed increased expression with increasing cellular proliferatin as shown by Ki-67 staining.

In conclusion, whilst pLIV-1 and pS2 levels appear to be associated with estrogen responsive pathways, pSyd3 and 8 are more closely linked to cellular proliferation. Since an increase in proliferation is associated with a loss of hormone sensitivity (Nicholson *et al.*, 1991, *Eur. J. Cancer*, **27**, 908-913) the increased levels of pSyd3 and 8 may play a role in the transition from endocrine responsive to unresponsive states.

**Oestrogenic and anti oestrogenic actions of tamoxifen analogues in rat mammary tumours *in vitro* and *in vivo***

A.L. Jarrett & S.A. Eccles

*Institute of Cancer Research, Sutton, Surrey, UK.*

Although clearly of great clinical value in the treatment of breast cancer, Tamoxifen has been shown to be a partial oestrogen agonist both *in vitro* and *in vivo*.

Using cell lines and clones of oestrogen-induced, and spontaneously arising hormone-responsive rat mammary tumours, we have compared the oestrogenic and anti-oestrogenic effects of Tamoxifen and two analogues. This has been achieved by assaying drug action under conditions of physiological hormone levels (antagonist assay) or of oestrogen deprivation (agonist assay).

*In vitro*, cell lines were selected which showed inhibition of growth in the presence of phenol-red free medium and DCC stripped FCS. Under these conditions, both Tamoxifen and two analogues (Iodo-tamoxifen and Pyrolidino-iodotamoxifen) were able to stimulate growth, as did oestradiol. In the presence of DMEM + 10% FCS (normal growth conditions) Tamoxifen inhibited cell growth and both analogues gave consistently better results.

*In vivo* tumours were implanted into OOX female rats. Cell lines were chosen which, under these conditions, would not develop without additional oestrogen. Groups of rats were dosed with TAM compounds at 2.4 mg/kg. Again we were able to show similar oestrogenic effects of all 3 compounds, although in normal animals, the two analogues have shown greater inhibitory effects on tumour growth than the parent compound.

These models offer a rapid and clinically relevant tumour assay system in which to screen separately for oestrogenic and anti-oestrogenic actions of new compounds.

### C-erb-2 overexpression in human breast carcinoma cell lines

D. Hollywood & H.C. Hurst

*ICRF Oncology Group, Cyclotron Building, Hammersmith Hospital, Du Cane Road, London W12 0HS, UK.*

This study investigates the mechanisms controlling the overexpression of *erb*B-2 mRNA in human breast carcinoma cell lines.

*erb*B-2 mRNA levels and gene copy number have been examined by Northern Hybridisation, RNA slot blotting, quantitation of *erb*b-2 mRNA half-life and Southern Hybridisation in a series of immortalised mammary epithelial cell lines (B—HBL100, MTSV1.7, MRSV2.1, MRSV2.4) and human breast carcinoma cell lines (ZR75-1, BT483, MDA MB 175, SKBR3). Malignant cell lines which exhibit 4-8 fold *erb*B-2 mRNA overexpression from the single copy gene have been identified.

The level of *erb*B-2 transcription was examined by nuclear run-on assays. Comparison of a non-tumourigenic cell line with baseline *erb*B-2 expression and malignant cell lines with *erb*B-2 overexpression has demonstrated a 3-fold increase in *erb*B-2 transcription in the malignant cell type.

Currently transcriptional control of the *erb*B-2 promoter in both types of cell line is being examined by short-term transfection of a series of *erb*B-2 promoter-CAT reporter gene constructs. Our results will be presented.

### Introduction of oncogenes into the mouse mammary gland using transplantation

J.M. Bradbury, S.E. Hiby, H. Sykes & P.A.W. Edwards

*Department of Pathology, University of Cambridge, Tennis Court Road, Cambridge, CB2 1QP.*

We have developed a method for expressing genes specifically in mouse mammary epithelium *in vivo*. Normal epithelial cells are isolated from an adult mouse and maintained in culture for four days. During this time, genes are introduced into the cells using non-replicating retroviruses. The altered cells are transplanted into the mammary fat-pad of another mouse from which all the endogenous epithelium has been removed by surgery (a cleared fat-pad). The transplanted cells reconstitute an epithelial 'tree' in which some of the cells express the introduced gene. This is a versatile model for studying growth, development and tumourigenesis in the mammary gland as a wide variety of genes can be introduced and their effect on the epithelial cells determined in their natural tissue environment.

The system has been used to investigate the effect of several oncogenes on the growth and development of the mammary gland. Introduction of the v-*myc* oncogene alone led to a mild hyperplasia in the gland where the epithelial ducts packed more closely than usual (Edwards, Ward & Bradbury, 1988, *Oncogene*, **2**, 407). v-Ha-*ras* alone seemed to inhibit the growth of transplants as many mammary glands into which v-Ha-*ras*-containing cells were transplanted were devoid of epithelial growth. In those glands where there was some growth it was often abnormal, resembling hyperplastic alveolar nodules, a preneoplastic lesion of mouse mammary gland. When both oncogenes were introduced simultaneously tumours resulted in a high percentage of the animals (Bradbury, Sykes & Edwards, 1991, *Int. J. Cancer*, **48**, 908). Other oncogenes shown to have effects on the growth pattern include *int-1* which caused vigorous hyperplasia in which the gland was packed with epithelium showing sidebranching like early pregnancy.

### The role of chromosome 17 in epithelial ovarian cancer

S.G.H. Russell[1], M. Murphy[2], D. Bell[2], P. Harkin[2], R.J. Atkinson[2], W.S. Lowry[2] and I. Hickey[3]

*[1]Department of Medical Genetics, [2]Department of Oncology, [3]School of Biology & Biochemistry, The Queen's University of Belfast, N. Ireland.*

Epithelial ovarian cancer accounts for approximately 5% of all new cancers in the United Kingdom each year.

We have used allele loss studies to indicate areas of the genome in which tumour suppressor gene inactivation may contribute to this disease.

In a preliminary investigation of 20 tumours, we described significant rates of allele loss from at least two loci on chromosome 17[1]. Approximately 50% of tumours showed loss from the p53 locus (17p13) while 75% showed loss with the probe pTHH59 (17q23-25).

We have now extended this investigation to a bank of 80 tumours and related allele loss at these two loci to histopathological type and stage of disease. Allele loss at p53 (17p13) was observed in serous, mucinous and endometrioid tumours but was more prevalent in stage III/IV disease than stage I/II. Allele loss in the region of the probe pTHH59 17q23-25 was also observed in the three histological subtypes and was high in both early and late stage disease.

We have used additional 17q probes to define the region of the loss on this chromosome arm. Partial allele losses were observed in 11 cases. The results are consistent with a target gene mapping to the region extending between the markers pTHH59 (17q23-25) and p05626 (17q25).

#### Reference

RUSSELL & Others (1990). *Oncogene*, **5**: p1581-83.

### FGF activity in conditioned medium derived from human breast tissues

A. Yelland, Y.A. Luqmani, J. Smith & R.C. Coombes

*CRC Laboratories, Department of Medical Oncology, Charing Cross Hospital, Fulham Palace Road, London W6 8RF.*

A number of putative growth factors (GFs) have been implicated in the maintenance and progression of breast cancer. We have investigated the presence of fibroblast growth factor-like (FGF-like) substances released from breast tissues and cultured cell lines into serum free conditioned medium after 24 and 72 h using the NR6 bioassay.

After 24 h the conditioned medium (CM) from cancers fell into two distinct groups, one third (15/45) were found to contain a high level of NR6 growth promoting activity, whilst the other two thirds had low level activity. The tumours with a high level of activity were associated with vascular invasion (p = 0.04). However, after a further 48 h, the CM from all cancers (n = 52) contained only low level activity. In twenty-two cases this activity was found to be heat stable and these cancers were significantly more likely to have metastasised to the regional lymph nodes ($p = 0.01$).

CM (72 h) from adjacent-histologically-normal (AHN) tissue was obtained in a similar manner to that for the cancers. The growth promoting activity for both cancer and AHN tissue was characterised using heparin affinity chromatography, neutralising antibodies and western blotting. We found evidence of both acidic and basic FGF-like activity. CM from two cell lines also contained a peptide co-migrating with authentic bFGF on SDS PAGE and which was detected by antibodies to bFGF on western blotting.

These results suggest that both normal and malignant breast tissues contain acidic and basic FGFs and that these appear to be released into the extracellular environment.

**Endothelin is a paracrine mitogen for human breast stromal cells**

K.V. Patel & M.P. Schrey

*Unit of Metabolic Medicine, Chemical Pathology and Clinical Endocrinology, St. Mary's Hospital Medical School, London W2 1PG.*

Human breast cancer cells have been recently reported to produce endothelin (ET)-1 (Kusuhara *et al.*, *Cancer Res.*, **50**, 3257, 1990). The function and regulation of ET-1 in the neoplastic breast is unknown. The aim of the present study is to assess the potential of ET-1 to act as a paracrine mitogen for human breast stromal cells. We have also monitored the ability of various hormones and growth factors to regulate ET-1 production by human breast cancer cells. ET-1 production by T47D cells was measured by radioimmunoassay and $[^3H]$ thymidine incorporation into human breast fibroblast DNA was measured in response to ET-1 and insulin-like growth factor 1(IGF-1).

Bombesin (0.1 $\mu$M) and cortisol (1 $\mu$M) stimulated maximal respective increases in ET-1 release to 580% and 369% of basal values ($7.2 \pm 0.4$ fmol/$10^6$ cells) after 6 h. The responses to cortisol and bombesin were additive. The response to bombesin was dose-dependent with an $ED_{50}$ around 1 nM and was inhibited by the receptor antagonist [$Leu^{13}$-$\psi$-$CH_2NH$-$Leu^{14}$] bombesin. ET-1 (10 nM) and IGF-1 (10 ng/ml) stimulated modest separate increases in DNA synthesis in human breast fibroblasts of 35% and 71% respectively, but together exhibited a strong synergistic response to 905% of control values. In the presence of IGF-1, significant increases in DNA synthesis were observed in response to 10 pM ET-1. This *in vitro* study demonstrates the potential for bombesin and glucocorticoid to regulate ET-1 production in human breast cancer cells, which may in turn have a paracrine influence on neighbouring stromal cell function.

This work is supported by the Cancer Research Campaign, UK.

**The B cell surface antigen, CD19, is involved in signalling entry into apoptosis**

C.D. Gregory & A.E. Milner

*Department of Immunology, The Medical School, Birmingham, B15 2TT.*

The term 'apoptosis' describes a genetically-controlled program of events which culminates in the self-destruction of cells. The regulation of apoptosis is known to determine the fate of a variety of cell types and inappropriate suppression of the process could contribute significantly to oncogenesis. B lymphocytes have the potential to enter apoptosis at stages of their differentiation when control is required over numbers of rapidly proliferating, genetically altered cells. These stages are: 1) in the bone marrow during initial immunoglobulin gene rearrangements, and 2) in the germinal centres of lymphoid tissue, during affinity maturation of antibody response. Our studies have recently shown (Nature, 349, **612,** 1991) that Burkitt lymphoma (BL) cell lines which retain the germinal centre B cell phenotype of the original tumour cells readily enter apoptosis, whereas isogenic lines expressing Epstein-Barr virus latent proteins do not. Using this model system, we now show that BL cells can be triggered into apoptosis through antibody-mediated ligation of their cell surface CD19 antigen. CD19, a member of the immunoglobulin supergene family, is a 'pan-B' cell marker, being expressed very early in B cell differentiation and retained until the terminal plasma cell stage. Its function in signalling entry into apoptosis therefore has important implications for B cell physiology. Our studies further indicate that susceptibility to CD19-induced apoptosis is limited to a narrow window of B cell differentiation and is significantly reduced following expression of the *bcl*-2 oncoprotein.

**Ras mutations as a prognostic indicator in malignant melanoma**

D.C.Lewis[1],V.K. Shukla[1], N. Warren[2], R.A. Padua[2] & L.E. Hughes[1]

*Departments of Surgery[1] and Haematology[2], University of Wales College of Medicine, Heath Park, Cardiff CF4 4XN.*

We have used the polymerase chain reaction and oligonucleotide hybridisation to detect point mutations of N, H, and K RAS in benign and dysplastic naevi, primary and metastatic melanoma.

Mutations were observed in 6 out of 26 naevi (23%), 9 out of 42 primary tumours (22%) and 9 out of 28 metastatic melanomas (32%), with K12 being the most frequently identified mutation. Of the 22 primary melanomas with lymph node involvement, there were 9 mutations (41%), each occurring in a separate patient. There were no patients in which the mutation was maintained from the primary to the secondary tumour. Of these 22 patients, those with a mutation at some stage of the disease had significantly reduced survival times of 25 months compared to 54 months in those without mutations ($p < 0.05$).

In the naevi all 6 mutations were found in the 9 removed following a significant increase in size, the presence of RAS mutations correlating strongly with clinical growth ($p < 0.001$). No correlation was found between age, sex, site, tumour thickness or the predisposition to metastatic spread and RAS mutations.

It appears that RAS mutations correlate strongly with growth in melanocytic lesions and tend to be associated with aggressive disease with a poor prognosis.

**The E5 gene of human papillomavirus type 16 can co-operate with the epidermal growth factor receptor**

R.J. Jewers, B. Kell & J.M. Best

*The Richard Dimbleby Laboratory of Cancer Virology, St Thomas' Hospital, London SE1 7EH.*

Human papillomavirus type 16(HPV-16) is strongly associated with anogenital neoplasias. It is established that two early genes, E6 and E7, encode oncoproteins that interact with the cellular tumour suppressors p53 and pRB, respectively. The function of another early gene product, E5, has only recently been determined (Jewers *et al.*, *Eur. J. Cancer*, in press).

We have expressed the HPV-16 E5 gene in murine fibroblasts and have shown anchorage independent growth and a loss of contact inhibition, both indicative of transformation. Exogenous epidermal growth factor (EGF) increases the frequency and size of colonies formed in soft agarose by HPV-16 E5 transfected 3T3 cells. In addition, HPV-16 E5 has a mitogenic effect on serum starved cells that is stimulated by exogenous EGF, with a resulting upregulation of the *c-fos*

gene. This suggests that HPV-16 E5 acts by increasing intracellular signals from the EGF receptor to the nucleus.

**Expression of steroid and epidermal growth factor receptors in MCF-7 cells resistant to multiple drugs**

R.D.H. Whelan, S. McClean & B.T. Hill

*Laboratory of Cellular Chemotherapy, Imperial Cancer Research Fund, London WC2A 3PX.*

Human breast tumours which are steroid receptor negative and express increased levels of epidermal growth factor receptor (EGFR) are associated with poor prognosis. Altered expression of these receptors has also been associated with the development of multidrug resistance (MDR) (Vickers *et al., Molecular Endocrinol.,* **2:** 886-892, 1988). Since drug resistance can be expressed following exposure of tumour cells not only to antitumour drugs but also to X-irradiation we have therefore determined the levels of oestrogen receptor (ER), progesterone receptor (PR) and epidermal growth factor receptor (EGFR) in a series of vincristine (VCR) resistant MCF-7 sublines derived following exposure to drug (MCF-7/VCR), alternate drug and X-ray treatments (MCF-7 VCR/DXR) or to fractionated X-irradiation (MCF-7 DXR/10). These sublines exhibited resistance to VCR (4- to 14-fold) and etoposide (2- to 3-fold). In contrast to X-ray pretreated cells MCF-7/VCR sublines expressed the classical MDR phenotype including cross-resistance to adriamycin and overexpression of P-glycoprotein. This expression of MDR was associated with a loss of detectable levels of ER and PR and elevated levels of EGFR (10-fold) determined by ligand binding assays. However, MCF-7/DXR10 cells retained parental cell levels of ER ($73 \pm 14$ fmoles/mg protein), PR ($215 \pm 20$) and EGFR ($7 \pm 1$). In addition, X-ray pretreated cells also proved sensitive to oestrogen and 4-OH tamoxifen and expressed similar levels of the oestrogen regulated pS2 peptide to parental cells after oestradiol stimulation. These studies indicate that ER, PR and EGFR are not consistently modified in cell lines which expressed resistance to multiple drugs.

**The relationship between expression of epidermal growth factor receptors by tamoxifen resistant and oestrogen independent breast cancer cell lines and response to epidermal growth factor**

B. Long, B.M. McKibben*, M. Lynch & H.W. van den Berg

*Departments of Therapeutics and Pharmacology and Medicine*, The Queen's University of Belfast, N. Ireland.*

Positive epidermal growth factor receptor (EGFR) status of primary human breast cancer has been reported to be a powerful indicator of poor prognosis and intrinsic resistance to hormonal therapy. In vitro experiments using cell lines from a number of sources have indicated no clear relationship between EGFR expression and response to EGF or transforming growth factor α. We have previously reported that tamoxifen resistant and oestrogen independent/tamoxifen sensitive variants of the ZR-75-1 human breast cancer cell line express markedly altered levels of EGFR (Long *et al., Br. J. Cancer,* **63,** Supplement XIII, 60, 1991). In this study we have examined the proliferative response of these cell lines to EGF under a variety of culture conditions. ZR-75-1 cells express $4,340 \pm 460$ EGFR sites/cell and failed to respond to EGF in serum-containing culture conditions. In serum-free medium (SFR) containing insulin ($5 \times 10$–7M) and oestradiol (10–8 M) cells proliferated more slowly but again failed to demonstrate a consistent response to EGF. The same results were obtained for the tamoxifen resistant line ($14,723 \pm 2116$ EGFR sites/cell) and the oestrogen independent line ($803 \pm 161$ EGFR site/cell). In SFM lacking insulin or oestradiol ZR-75-1 cells failed to proliferate but showed a marked dose-dependent growth stimulatory response to EGF (0.01-100 ng/ml). In contrast the variant lines continued to proliferate in the absence of insulin or oestradiol and exogenous EGF was without effect. Failure of the oestrogen independent line to respond did not appear to be the result of receptor occupation by autocrine growth factor(s) as neither acid washing or suramin treatment uncovered further receptors. Whilst minimal receptor expression might explain the failure of this line to respond to EGF an elevated level of EGFR typified by the tamoxifen resistant variant did not confer sensitivity to exogenous ligand.

**Autocrine/paracrine growth regulation by EGF and TGF-α in human ovarian adenocarcinoma cell lines**

A.J. Crew, J.M.S. Bartlett, W.N. Scott, E.P. Miller, G.J. Rabiasz, S.P. Langdon & W.R. Miller

*ICRF Medical Oncology Unit, Western General Hospital, Edinburgh EH4 2XU.*

In primary ovarian cancers, EGF receptors have been detected in 40–70% of tumours and EGF and TGF-α detected in 38% and 85% respectively. This coincidental expression suggests that EGF and TGF-α may regulate growth of ovarian cancer cells via an autocrine or paracrine pathway.

In order to test this hypothesis of growth regulation via EGF/TGF-α, two human ovarian adenocarcinoma cell lines, PEO4 and PEO14 have been studied as model systems. Using an RNAse protection assay, the mRNA for TGF-α has been detected in the PEO4 cell line, but is absent in the PEO14 cell line, whereas the EGF protein has been detected in the conditioned media of both cell lines by RIA, providing evidence for the production of the factors. EGF receptors have been identified on the two cell lines by immunohistochemical techniques and ligand binding. In addition, the growth of both cell lines is stimulated by EGF and TGF-α. EGF causes a rapid down-regulation of EGF receptors in both cell lines. Oestrogen also produces a decrease in EGF receptor level in the oestrogen-sensitive oestrogen receptor (ER)-positive cell line, PEO4, although this is less pronounced and delayed compared to the effects of EGF. No changes in the EGF receptor level were produced by oestrogen in the ER-negative PEO14 cell line. It is suggested that since all the components of an EGF/TGF-α autocrine/paracrine loop are present on both cell lines, these may be potential important growth regulatory pathways in ovarian cancer, and in tumours which are ER-positive, may be influenced by oestrogen.

**A comparison of two methods for the detection of epidermal growth factor receptor**

D.J. Hastrich, J.M. Dunn, P. Newcomb, S.Nicholson & J.R. Farndon

*Department of Surgery, Bristol Royal Infirmary, Bristol BS2 8HW.*

Controversy continues about the value of Epidermal Growth Factor receptor (EGFr) as a prognostic indicator in women with breast cancer. It is difficult to compare one study with another when the methods used vary in basic method. We have ascertained EGFr status on 100 tumours by competitive radioligand assay and by immunohistochemistry using the monoclonal antibody to EGFr R1 (Amersham). There was no corelation between tumour grade or size and EGFr status with either method. Of 54 tumours positive on the assay 40 were positive on immunohistochemistry and of 46 tumours negative on assay. 41 were negative on R1 staining; there was a strong correlation between the two methods. If the assay is assumed to be the gold standard, immunohistochemistry has a specificity of 74% and a sensitivity of 89%. Only long-term follow-up of these patients will determine whether this fall in sensitivity adds or detracts from the prognostic value of EGFr.

**Inhibition of non-small cell lung cancer (NSCLC) cell proliferation by intervention in the EGF receptor pathway**

R. Hoffman & J. Donaldson

*Department of Clinical Oncology, MRC Centre, Hills Road, Cambridge CB2 2QH.*

Autocrine growth stimulation by TGFα is implicated in the proliferation of NSCLC cells. Intervention in this process therefore represents a potential target for anti-cancer therapy. We examined EGF receptor expression in 4 cell lines derived from patients diagnosed with NSCLC (L23P and its multidrug resistant variant L23AR, MOR and BEN). Scatchard analysis of competition curves between EGF and [$^{125}$I]EGF indicated the presence of a single class of EGF receptor in L23P, L23AR and MOR cells (Kd values 7–8 nM). BEN cells did not contain detectable levels of EGF receptors. Spent medium from L23P cells competed with [$^{125}$I]TGFα for binding to A431 cells, indicating that an autocrine loop involving TGFα and the EGF receptor exists in these cells. The anti-proliferative effects of EGF/TGFα pathway antagonists were assessed by the MTT assay following a 5d exposure. Growth of all the cell lines in serum was inhibited by suramin (IC50 values of 0.15–0.24 mM). The EGF receptor tyrosine kinase inhibitor 3,4 dihydroxybenzylidene cyanoacetamide inhibited growth of the cell lines in serum in decreasing order of potency MOR > L23AR > L23P > BEN (IC50 values 37, 47, 62 and 118 μM respectively). 3,4 dihydroxybensylidene malononitrile, which is a 4-fold less potent inhibitor of the EGF receptor kinase, inhibited all the cell lines with similar potency. There was little growth inhibition after 1d. Growth inhibition by the tyrosine kinase inhibitors of cells grown in a defined medium in the absence of exogenously added growth factors was similar to growth inhibition in serum. The weak EGF receptor tyrosine kinase inhibitor 2 amino-4-(4-hydroxyphenyl)-1,1,3-tricyanobuta-1,3-diene caused less than 50% inhibition of all cell lines at concentrations up to 200 μM. These data indicate that agents which inhibit signal transduction by the EGF receptor may be useful anti-proliferative agents in EGF receptor-positive NSCLC including cells expressing the MDR phenotype.

**Rat mabs to the human EGFR receptor for therapeutic application**

H. Modjtahedi, J. Styles, J. Sandle, G. Box, S. Eccles & C. Dean

*Institute of Cancer Research, Belmont, Sutton, Surrey, UK.*

The receptor for epidermal growth factor (EGFR) and transforming growth factor α (TGFα) are often overexpressed by squamous cell and other carcinomas and these receptors may therefore form targets for antibody directed therapy. We have produced a series of rat mAbs that recognise the external domain of the human EGFR using as immunogen either the head and neck carcinoma LICR-LON HN5 or the breast carcinoma cell line MDA-MB 468. All of these antibodies immunoprecipitate the 170kD EGFR and 10/15 antibodies inhibit the binding of $^{125}$I-EGF to HN5, EJ and MDA-MB468 cells. Competition analyses show that the latter mAbs bind to three distinct epitope clusters on the EGFR. These antibodies also block the EGF induced growth of fibroblasts and more importantly inhibit the growth *in vitro* of several squamous cell carcinoma cell lines (HN5, HN6, MDA-MB 468 and A431). The extent of growth inhibition is related to the number of receptors expressed and is greatest with HN5 cells ($5 \times 10^6$ EGFR/cell). Flow cytometric cell cycle analyses using HN5 cells show that after incubation in the presence of two of the mAbs (ICR16, rat IgG2a and ICR62, rat IgG2b) the cultures show a marked decrease in cells in the S and the G2 phases. At concentrations above 5nM these antibodies completely block the growth of HN5 cells *in vitro*. Antibodies ICR16 and ICR62 effectively suppress the growth of HN5 tumour xenografts in athymic mice when antibody treatment (total dose 3.0 mg/mouse) was started at the time of tumour inoculation. Tumour growth was suppressed also when treatment with these antibodies was delayed until 9 days after tumour inoculation. The greater efficiency of antibody ICR62 *in vivo* compared to its activity *in vitro* relative to ICR16 suggest that the former may also be acting via interation with immune effector cells and/or host complement.

**The *c-erbB2 p185* as a target for antibody therapy in breast cancer**

S. Eccles, J. Styles, M. Valeri, A. Bakir, H. Modjtahedi, J. Sandle & C. Dean

*Institute of Cancer Research, Sutton, Surrey, UK.*

The overexpression of receptors for epidermal growth factor by squamous cell carcinomas and of the related proto-oncogene c-erbB-2 (p185) by adenocarcinomas may provide useful targets for specific antibodies. The product of the c-erbB-2 gene is overexpressed in some 20% of breast and ovarian carcinomas and patients whose tumours overexpress the c-erbB-2 P185 have a poor prognosis. We have prepared a series of rat monoclonal antibodies that bind to one of five non-overlapping epitope clusters on the external domain of the c-erbB-2 P185. All of the antibodies immunoprecipitate the 185 kD protein and none binds to the related EGF receptor. One of these antibodies (ICR12, rat IgG2a) binds to p185 in Western blots and stains formol-saline fixed paraffin embedded material. This antibody, which is of high affinity (0.2nM) has been found to localise specifically and stably to xenografted breast and ovarian tumours in nude mice (up to 20% injected dose/g) when labelled with Indium-111, Iodine-125 or the positron emitter Iodine-124. Under these conditions tumour uptake of a control, isotype matched antibody was less than 4% i.d./g. Tumour xenografts of 5 mm diameter were successfully imaged at 24,48 and 120 h using the RMH-ICR MUP-PET camera. ICR12 labelled with Iodine-124 is currently being evaluated in an imaging trial in patients with breast cancer at the Royal Marsden Hospital, Sutton.